# Priority Micronutrient Density of Foods for Complementary Feeding of Young Children (6–23 Months) in South and Southeast Asia

**DOI:** 10.3389/fnut.2021.785227

**Published:** 2021-12-21

**Authors:** Flaminia Ortenzi, Ty Beal

**Affiliations:** ^1^Consultant, Geneva, Switzerland; ^2^Global Studies Institute, University of Geneva, Geneva, Switzerland; ^3^Global Alliance for Improved Nutrition (GAIN), Washington, DC, United States; ^4^Department of Environmental Science and Policy, University of California, Davis, Davis, CA, United States

**Keywords:** nutrient density, complementary feeding, South Asia, Southeast Asia, micronutrient deficiencies, 6–23 months, animal-source foods, dark green leafy vegetables

## Abstract

**Background:** Given their high nutrient requirements and limited gastric capacity, young children during the complementary feeding period (6–23 months) should be fed nutrient-dense foods. However, complementary feeding diets in low- and middle-income countries are often inadequate in one or more essential micronutrients. In South and Southeast Asia infants' and young children's diets are commonly lacking in iron, zinc, vitamin A, folate, vitamin B_12_, and calcium, hereafter referred to as priority micronutrients.

**Objective:** This study aimed to identify the top food sources of priority micronutrients among minimally processed foods for complementary feeding of children (6–23 months) in South and Southeast Asia.

**Methods:** An aggregated regional food composition database for South and Southeast Asia was built, and recommended nutrient intakes (RNIs) from complementary foods were calculated for children aged 6–23 months. An approach was developed to classify foods into one of four levels of priority micronutrient density based on the calories and grams required to provide one-third (for individual micronutrients) or an average of one-third (for the aggregate score) of RNIs from complementary foods.

**Results:** We found that the top food sources of multiple priority micronutrients are organs, bivalves, crustaceans, fresh fish, goat, canned fish with bones, and eggs, closely followed by beef, lamb/mutton, dark green leafy vegetables, cow milk, yogurt, and cheese, and to a lesser extent, canned fish without bones.

**Conclusions:** This analysis provided insights into which foods to prioritize to fill common micronutrient gaps and reduce undernutrition in children aged 6–23 months in South and Southeast Asia.

## Introduction

The first 2 years of a child's life represent a “critical window” for the achievement of optimal growth and health, for which adequate nutrition is an essential prerequisite. The reversion of stunting becomes difficult after a child reaches 2 years of age, further demonstrating the crucial importance of intervening during this time. This is also the age when growth impairments, deficiencies in certain micronutrients, and common childhood illnesses are most likely to happen (1). In particular, according to the World Health Organization (WHO) and other available literature, the highest occurrence of stunting in low- and middle-income countries is registered during the complementary feeding period, which corresponds to 6–23 months of age (2, 3).

Africa and Asia are the regions where the largest proportion of malnourished children under 5 years of age live. Together, they account for more than nine out of ten of all stunted children and more than nine out of ten of all wasted children globally (4). Within the Asian region, South Asia is home to about a quarter of the world's children under 5 years and has the highest percentages and numbers of stunted (30.7%, *n* = ~54 million) and wasted (14.1%, *n* = 25 million) children (5), closely followed by Southeast Asia which has the second highest percentages and numbers of stunted (27.4%, *n* = ~15 million) and wasted (8.2%, *n* = 4.6 million) children (5). Inadequate diets during the complementary feeding period are among the key determinants of the child-stunting and wasting crisis in South and Southeast Asia (2, 6).

The WHO defines complementary feeding as the transition time when other foods and liquids, along with breast milk, are needed to meet a child's nutrient requirements and are gradually introduced in their diet (1, 3). Infants and young children 6–23 months of age have very high nutrient requirements per unit body weight, because of their rapid growth and development rates. Continued breastfeeding can significantly contribute to meeting their nutrient needs, but has to be complemented with a variety of nutritionally adequate and safe family foods (1). Children aged 6–23 months have a limited gastric capacity and can only consume small quantities of food, therefore, complementary foods should have high nutrient density (amount of each nutrient per 100 kcal of food) (1, 2). Unfortunately, in practice this is often not the case, and diets of infants and young children are often not sufficiently diverse and nutrient dense and are lacking in one or more essential micronutrients, especially in low- and middle-income countries, but also in high-income countries [e.g., iron and zinc in the US; (1, 2, 7)].

In particular, in South and Southeast Asia important micronutrient gaps were identified in infants' and young children's diets (7–10). The micronutrients most commonly known to be lacking and those of highest public health significance in the two regions are iron, zinc, vitamin A, folate, vitamin B_12_, and calcium, hereafter referred to as priority micronutrients (7–10). Among the main drivers of micronutrient malnutrition in infants and young children living in South and Southeast Asia are the following: (1) the low micronutrient density and lack of diversity of complementary foods, with most children aged 6–23 months having a primarily cereal-based complementary feeding diet and not consuming the minimum recommended number of food groups each day (6, 7, 10–12); and (2) the inappropriate marketing of nutritionally inadequate, often ultra-processed, complementary foods, and beverages that are promoted as suitable for this age group (11).

Given the crucial importance of adequate nutrition in infancy and early childhood and its impact on children's present and future lives and on societies as a whole, improving the overall quality and diversity of infants' and young children's diets is critical to the achievement of the Sustainable Development Goals (SDGs) (6). The purpose of this study is to identify the top food sources of priority micronutrients, among minimally processed, inherently nutrient-dense foods, to support efforts to reduce micronutrient malnutrition among complementary fed children (6–23 months) in the South and Southeast Asian regions.

## Methods

### Calculating Recommended Nutrient Intakes

Based on a previously adopted approach to identify affordable nutritious complementary foods (7, 13), Recommended Nutrient Intakes (RNIs) from complementary foods for children aged 6–23 months were calculated from:

- The WHO and the Food and Agriculture Organization (FAO) recommendations (14) for calcium, zinc, and iron;
- The Institute of Medicine (IOM) recommendations (15) for folate, vitamin A, and vitamin B_12_

Average Energy Requirements (AERs) from complementary foods for children 6–23 months of age were calculated from the WHO and the United Nations Children's Fund (UNICEF) recommendations, accounting for average breast milk intake in developing countries (1, 3). The RNI is the intake level sufficient to meet the daily nutrient requirements of almost all individuals (97.5%) in a specific age and gender group (14); while the Average Requirement (AR) is the intake level that is adequate for half (50%) of the individuals in a given population group (16). We decided to use RNIs for the six included micronutrients rather than ARs because this study focuses on achieving micronutrient adequacy for individuals, not on estimating adequacy at the population level [(17); see Supplementary Material for a more detailed explanation of how RNIs and AERs from complementary foods were calculated].

### Building a Regional Food Composition Database for South and Southeast Asia

A regional food composition database, representative of the nutritional value of foods in South and Southeast Asia, was built^1^, including values for calories, phytate (18), and for the six identified priority micronutrients: vitamin A, folate, vitamin B_12_, calcium, iron, and zinc. Nutrient values were obtained from US Department of Agriculture (USDA) FoodData Central (FDC) (19) and from several South and Southeast Asian countries' food composition tables (FCTs) (20): Bangladesh, Indonesia, Laos, Vietnam, and Thailand. Values from FDC were included in the calculations of composite nutrient values, serving as a reference to ensure plausibility of values from the selected national FCTs and, on a few occasions, they were also used to replace missing values from other FCTs^2^. All foods were included in the forms typically consumed, which could be raw (e.g., fruits), cooked (e.g., meat and poultry, pulses), or a mix of both (e.g., vegetables). For foods typically consumed in their cooked form, but with values only available as raw in the included FCTs, weight yields and nutrient retention factors for different cooking methods were applied (21).

Foods presenting low nutrient density variance (e.g., pulses, refined grains) or likely to be targeted as a food group rather than individually in policy and programming (e.g., dark green leafy vegetables, cheese) were aggregated for analysis. For foods analyzed individually, nutrient values were obtained by calculating the medians of country-level composite values from the selected FCTs. Composite values were obtained by averaging nutrient values for different cooking methods (as well as raw foods, if applicable) and for different cuts of the animal in the case of meat and poultry. For aggregated food groups, nutrient values were calculated by averaging regional-level composite values from South and Southeast Asia and from FDC. Composite values were obtained by calculating the medians of nutrient values for several individual foods (e.g., spinach, kale, amaranth leaves, and others) within a given food group (e.g., dark green leafy vegetables), which were derived from multiple included FCTs located in the South and Southeast Asian regions.

The bioavailability of iron and zinc was accounted for in the analysis. Foods included in the regional food composition database were assigned to one of three levels of iron absorption: 20% for ruminant meat, 15% for all other animal-source foods (ASFs), and 10% for all plant-source foods (PSFs), based on the proportion of heme to non-heme iron contained (22). The following heme-iron percentages were assumed: 68% in ruminant meat, including beef (23–25), goat, and lamb/mutton (25, 26); 39% in pork (24, 25, 27–29); 26% in chicken (24, 25, 27–29), fish and seafood (24, 27–30), eggs and dairy (28); and 40% in all other meat, including offal (23, 28, 29). In addition, foods were categorized into four levels of zinc absorption: 44, 35, 30, and 26%, based on the amount of phytate contained in a portion equivalent to one-third of daily mass intake, assuming an energy density of 1.3 kcal/g^3^ and considering average energy requirements for a moderately active woman of reproductive age (16). A similar approach was previously used to build a global food composition database [(32); see Supplementary Material for a more detailed explanation of the approach adopted to develop the regional food composition database for South and Southeast Asia].

### Aggregate and Individual Priority Micronutrient Density Ratings

Foods were categorized into four levels of priority micronutrient density based on the portion (calories and grams) needed to achieve one-third (for individual ratings) or an average of one-third (for the aggregate rating) of RNIs from complementary foods for the six selected micronutrients (vitamin A, folate, vitamin B_12_, calcium, iron, and zinc). For the aggregate score, the Average Share of Recommended Intakes (*ASRI*) across the six micronutrients (*A*), for a given quantity of grams (*i*), of a given food (*j*), was calculated as:


$$\begin{array}{l} ASRI_{i,j}=\frac{1}{\left|A\right|}\underset{a\;\in \;A}{\sum }\min\left\{\frac{nutrient\text{\_}density_{a,j}\;*\;i}{recommended\text{\_}intakes_{a}},\;1\right\} \end{array}$$

A similar approach was previously used to identify nutrient-dense foods for infants and young children and for other population groups (7, 13, 32). As illustrated in the formula, each micronutrient's contribution was capped at 100% of RNIs, meaning that each micronutrient can contribute from 0% up to 50% of the overall score. This choice was made to ensure that foods would only be rated high if they were high in at least two out of the six micronutrients included in the analysis; and to prevent foods with very high densities of individual micronutrients from rating higher for providing amounts well above recommended intakes or even above upper limits. A similar approach was previously applied to identify top food sources of priority micronutrients for other population groups (32) and used to determine micronutrient-dense complementary foods (7, 13).

Foods were ranked according to the following thresholds on Average Requirements (ARs) for energy from complementary foods for children 6–23 months of age and hypothetical ARs for mass, assuming an energy density of 1.3 kcal/g [obtained by averaging the minimum and maximum composite energy densities of the sample complementary feeding diets for breastfed children developed by the World Health Organization (33)]:

- Very high: ≤ one-sixth of ARs for both energy and mass
- High: ≤ one-third of ARs for both energy and mass and < one-sixth of ARs for either energy or mass
- Moderate: ≤ one-third of ARs and > one-sixth of ARs for both energy and mass
- Low: > one-third of ARs for either energy or mass

The above thresholds were chosen by taking into consideration the assumed functional gastric capacity of children aged 6–23 months (30 g/kg body weight/d) and plausible amounts of complementary foods that they could consume at each meal, as well as meal frequency during the complementary feeding period (1, 34).

A slightly different approach was taken for milk (cow and goat), to account for the fact that mass is a less limiting factor for liquids than for solid foods in children 6–23 months of age. The same thresholds on energy and mass as for solid foods were applied, but instead of hypothetical ARs for mass, a maximum daily intake of milk of 400 g was assumed, which was considered plausible for children during the complementary feeding period based on a review of Food-Based Dietary Guidelines (FBDGs) from multiple countries (35, 36).

## Results

### Recommended Nutrient Intakes for Children 6–23 Months of Age

The AR for energy from complementary foods for children aged 6–23 months is 450 kcal/d, accounting for average breast milk intake in developing countries (1). In addition to energy requirements, breast milk contributes to the achievement of RNIs for breastfed infants and young children during the complementary feeding period to different extents depending on the nutrient considered (Table 1). For instance, vitamin A requirements are largely covered by breast milk, with only 20% needed from complementary foods. Folate, vitamin B_12_, and calcium requirements are also partially covered by breast milk intake, while for iron, the totality and for zinc, the near totality of RNIs need to be obtained through complementary foods. Moreover, recommended intakes for iron and zinc significantly vary depending on bioavailability: the lower the bioavailability level, the higher the RNIs, as larger amounts of iron and zinc are necessary to meet nutrient requirements.

**Table 1:** Recommended Nutrient Intakes (RNIs) for children 6–23 months of age.

**Nutrient**	**Total recommended nutrient intakes^c^**	**Proportion required from complementary foods^b^**	**Recommended nutrient intakes from complementary foods^e^**
Vit A (μg RAE)	367	0.2	73
Folate (μg DFE)	127	0.6	76
Vit B_12_ (μg)	0.8	0.7	0.5
Calcium (mg)	467	0.7	327
Iron^a^ (mg) 20%	3.5	1.0	3.5
Iron^a^ (mg) 15%	4.7	1.0	4.7
Iron^a^ (mg) 10%	7.0	1.0	7.0
Zinc^b^ (mg) R	2.8	0.9	2.5
Zinc^b^ (mg) SR	3.5	0.9	3.1
Zinc^b^ (mg) SU	4.1	0.9	3.7
Zinc^b^ (mg) U	4.7	0.9	4.2

*Recommended intakes for calcium, iron, and zinc from the World Health Organization and Food and Agriculture Organization of the United Nations (14). Recommended intakes for vitamin A, folate, and vitamin B_12_ from the Institute of Medicine (15)*.

a *Percentages (20, 15, and 10%) indicate the three levels of iron absorption considered in the analysis*.

b *Assuming 300 mg phytate/day and 44% absorption for refined (R) diets, 600 mg phytate/day and 35% absorption for semi-refined (SR) diets, 900 mg phytate/day and 30% absorption for semi-unrefined (SU) diets, and 1,200 mg phytate/day and 26% absorption for unrefined (U) diets*.

c *Total recommended intakes to be achieved through the combination of breast milk (or formula) and complementary foods*.

d *Assuming that the remaining proportion of recommended intakes would be provided by breast milk (or formula), based on Dewey (37)*.

e *Recommended intakes to be achieved through complementary foods only, accounting for breast milk intake*.

*RAE, retinol activity equivalent; DFE, dietary folate equivalent; R, refined; SR, semi-refined; SU, semi-unrefined; U, unrefined; Vit, vitamin*.

### Regional Food Composition Database for South and Southeast Asia

A regional food composition database for South and Southeast Asia was built, including a total of 36 individual foods and aggregate food groups, with values for energy, the six priority micronutrients, calculated iron and zinc absorption levels, and phytate (Table 2). While most aggregate food groups, such as grains and their products (both whole and refined), “other fruits” and “other vegetables,” present low nutrient density variance across included foods and across different countries' FCTs (Supplementary Table 2), some, such as dark green leafy vegetables (DGLVs) and fresh fish show greater nutrient density variance across included foods (Supplementary Tables 2, 4). For instance, spinach, amaranth leaves, and cassava leaves are more nutrient-dense than lettuce and cabbage. In the case of fresh fish, herring, carp, and mackerel (fatty fish) have higher nutrient values than sea bass and tilapia (lean fish). In addition, for a few foods and food groups, differences in the amounts of some nutrients were identified across the included FCTs. For example, values for folate in pulses and for vitamin A in beef liver are higher in USDA FDC than in South and Southeast Asian countries' FCTs, which may be due to different varieties, soil conditions, types of animal feed, culinary traditions, cooking methods and length, and/or the quality of sampling and analysis, including methods of mass spectrometry, conducted to develop the FCTs.

**Table 2:** Regional food composition database for South and Southeast Asia.

**Food (100 g)**	**kcal**	**Vit A (mcg RAE)**	**Folate (mcg DFE)**	**Vit B_**12**_ (mcg)**	**Calcium (mg)**	**Iron (mg)**	**Zinc (mg)**	**Iron Abs**	**Zinc Abs**	**Phytate (mg)**
Pulses	140	1	97	0	27	2.7	1.3	0.10	0.26	441
Whole grains^a^	158	0	15	0	13	1.7	1.1	0.10	0.26	443
Refined grains^a^	131	0	5	0	9	0.4	0.6	0.10	0.44	42
Whole grain products^a^	194	0	84	0	28	1.2	1.2	0.10	0.35	77
Refined grain products^a^	155	0	6	0	9	0.7	0.5	0.10	0.44	49
Millet	132	0	23	0	10	2.0	1.1	0.10	0.26	200
Nuts	581	1	80	0	76	4.3	3.1	0.10	0.26	646
Seeds	561	1	97	0	134	6.9	5.6	0.10	0.26	402
Starchy roots, tubers, and plantains	101	16	11	0	22	0.7	0.3	0.10	0.44	12
DGLVs	27	286	37	0	102	2.0	0.3	0.10	0.44	16
Vit A-rich fruits and veg, excl DGLVs	38	126	21	0	20	0.4	0.2	0.10	0.44	24
Other fruits, excl vit A-rich fruits	62	4	17	0	11	0.3	0.1	0.10	0.44	10
Other veg, excl DGLVs, and vit A-rich veg	24	15	15	0	19	0.5	0.3	0.10	0.44	11
Hen egg	157	164	45	1.1	43	1.5	1.8	0.15	0.44	0
Beef	273	3	3	1.7	10	2.5	6.3	0.20	0.44	0
Goat	112	0	4	1.2	12	2.8	3.9	0.20	0.44	0
Lamb/mutton	210	2	10	2.5	13	1.7	4.1	0.20	0.44	0
Pork	234	1	3	0.6	21	1.5	2.1	0.15	0.44	0
Chicken	197	17	4	0.2	10	0.8	1.3	0.15	0.44	0
Fresh cow milk	67	52	5	0.4	120	0.1	0.4	0.15	0.44	0
Cooked cow milk	61	32	5	0.5	113	0.1	0.4	0.15	0.44	0
Fresh goat milk	69	45	1	0.1	143	0.1	0.3	0.15	0.44	0
Yogurt	61	27	7	0.4	121	0.1	0.6	0.15	0.44	0
Cheese	358	224	19	0.9	691	0.4	3.3	0.15	0.44	0
Beef liver	151	6,166	226	60.4	10	7.7	4.3	0.15	0.44	0
Goat/lamb liver	229	7,637	237	81.1	9	9.2	6.8	0.15	0.44	0
Chicken liver	147	3,492	509	16.1	12	9.4	3.4	0.15	0.44	0
Pork liver	139	4,924	149	16.9	10	15.9	5.7	0.15	0.44	0
Heart^b^	128	5	15	6.1	9	5.3	3.3	0.15	0.44	0
Spleen^b^	149	0	4	5.0	13	38.7	3.5	0.15	0.44	0
Kidney^b^	104	86	53	14.7	12	5.6	2.7	0.15	0.44	0
Fresh fish^c^	120	9	15	4.0	30	3.1	2.1	0.15	0.44	0
Crustaceans	92	3	15	1.2	87	1.2	3.2	0.15	0.44	0
Bivalves	106	68	7	17.6	68	4.0	1.9	0.15	0.44	0
Canned fish, without bones	136	20	4	2.6	17	1.4	0.7	0.15	0.44	0
Canned fish, with bones	182	43	10	3.9	240	2.5	1.2	0.15	0.44	0

*All included foods and aggregate food groups are listed in the forms typically consumed, which could be raw, cooked, or a mix of both, depending on the food/food group considered*.

a *The terms “whole grains” and “refined grains” refer to cereals, such as wheat, rice, and barley, while the terms “whole grain products” and “refined grain products” refer to products obtained from cereal flours, such as breads, pasta, noodles, and vermicelli.*

b *From different animals, including beef, lamb, pork, and chicken*.

c *Includes different species of marine and freshwater fish*.

*Vit, vitamin; RAE, retinol activity equivalent; DFE, dietary folate equivalent; Excl, excluding; Veg, vegetables; Abs, absorption*.

### Aggregate Priority Micronutrient Density Scores of Foods for Children 6–23 Months of Age

**Figure 1:**
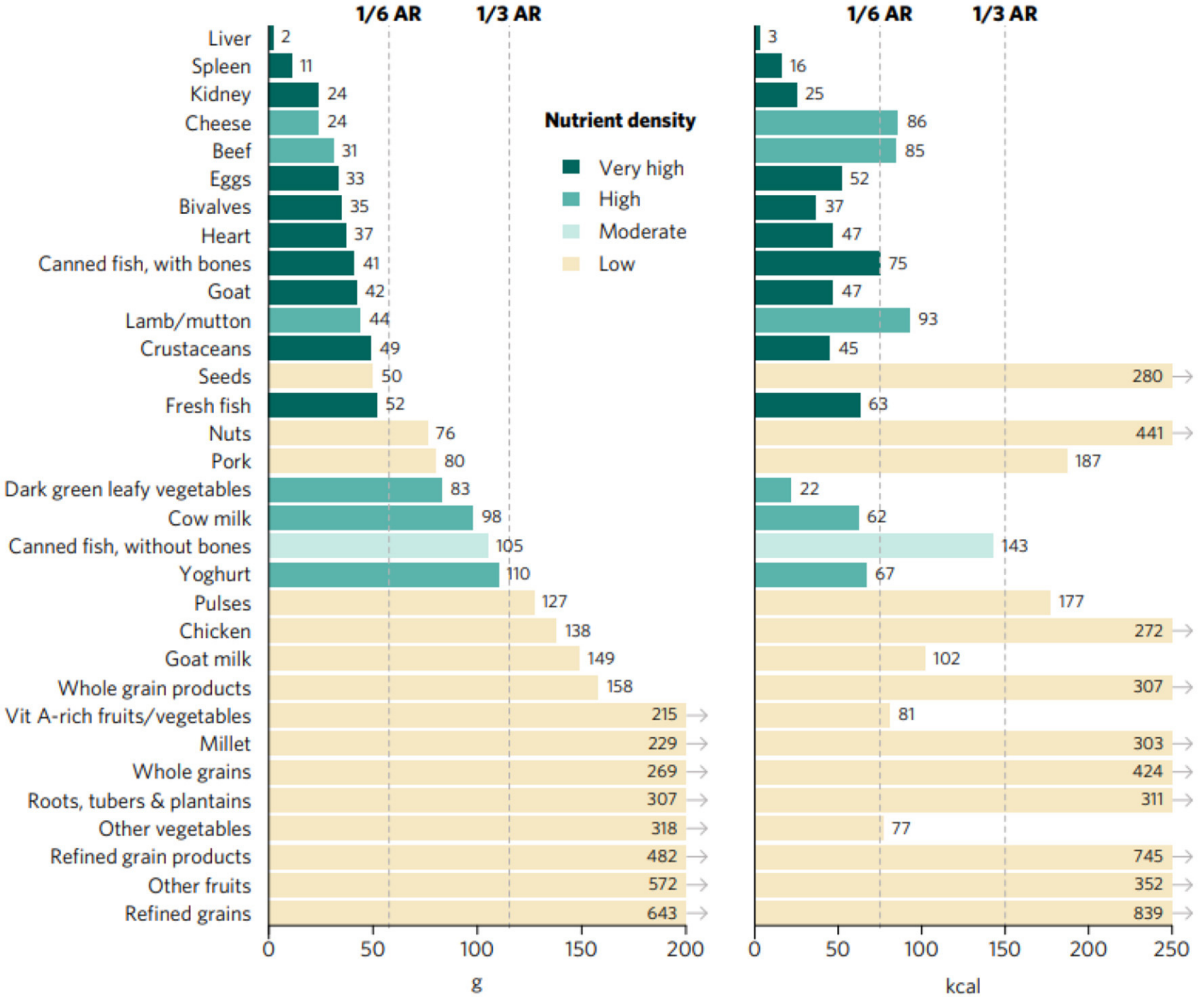
Portion sizes (calories and grams) needed to provide an average of one-third of recommended intakes from complementary foods of vitamin A, folate, vitamin B12, calcium, iron, and zinc for children aged 6–23 months. Each micronutrient's contribution to the aggregate score was capped at 100% of recommended intakes. Hypothetical average requirement (ARs) for mass were obtained by assuming an energy density of 1.3 kcal/g.

Portion sizes, expressed as calories and grams, required to achieve an average of one-third of RNIs from complementary foods of vitamin A, folate, vitamin B_12_, calcium, iron, and zinc for children aged 6–23 months range from <5 g and kcal for liver (from different sources—beef, goat/lamb, chicken, and pork) to more than 600 g and kcal for refined grain products (Figure 1). Foods presenting very high aggregate priority micronutrient density (referred to as “top sources” hereafter) are the following: organs, including liver, spleen, kidney and heart from beef, goat/lamb, chicken, and pork; bivalves (clams, mussels, and oysters); crustaceans; fresh fish, including different species of marine and freshwater fish; goat; canned fish with bones; and eggs. Foods with a high aggregate micronutrient density include beef, lamb/mutton, DGLVs, cow milk, yogurt, and cheese, followed by canned fish without bones which was rated as moderate. All other foods analyzed presented low aggregate priority micronutrient density, including some animal-source foods (goat milk, pork, and chicken) and several plant-source foods.

### Individual Priority Micronutrient Density Scores of Foods for Children 6–23 Months of Age

**Figure 2:**
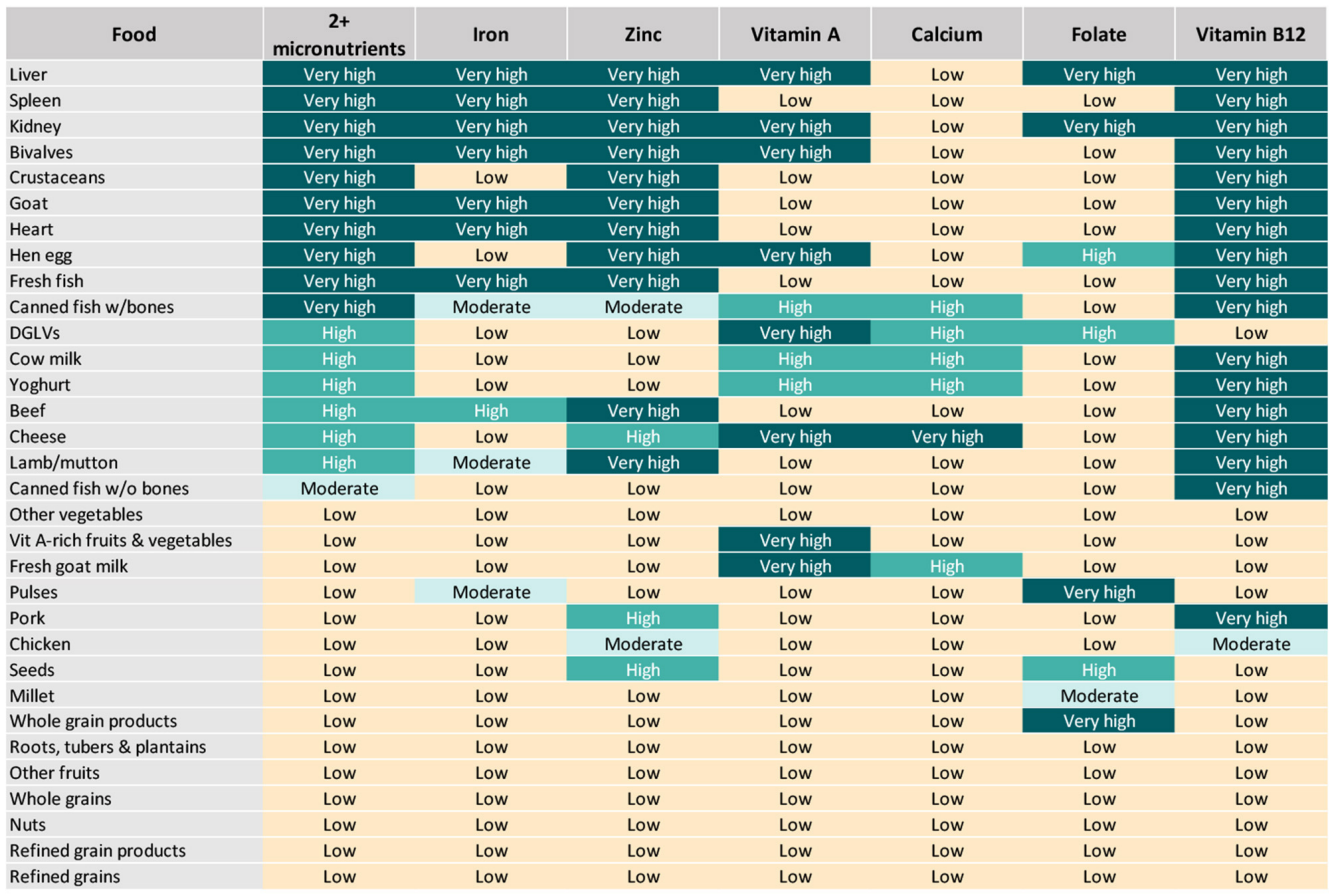
Aggregate and individual priority micronutrient density scores for children aged 6–23 months.

All analyzed foods scored low in at least one of the six priority micronutrients (Figure 2), exemplifying the importance of varied diets during the complementary feeding period, with a particular focus on the most nutrient-dense foods, to satisfy all nutrient requirements of infants and young children. For instance, liver and kidney were rated as very high in all included micronutrients except for calcium, for which they scored low. Most animal-source foods (besides chicken and canned fish without bones) and DGLVs scored very high or high in two or more micronutrients. Some plant-source foods presenting a low aggregate score were rated as very high or high in certain micronutrients. For example, vitamin A-rich fruits and vegetables scored very high in vitamin A; pulses and whole grain products scored very high in folate; and seeds scored high in zinc and folate. Others, such as starchy roots, tubers and plantains, refined grains and their products, “other vegetables,” and “other fruits” were not rated as very high or high in any of the priority micronutrients.

**Figure 3:**
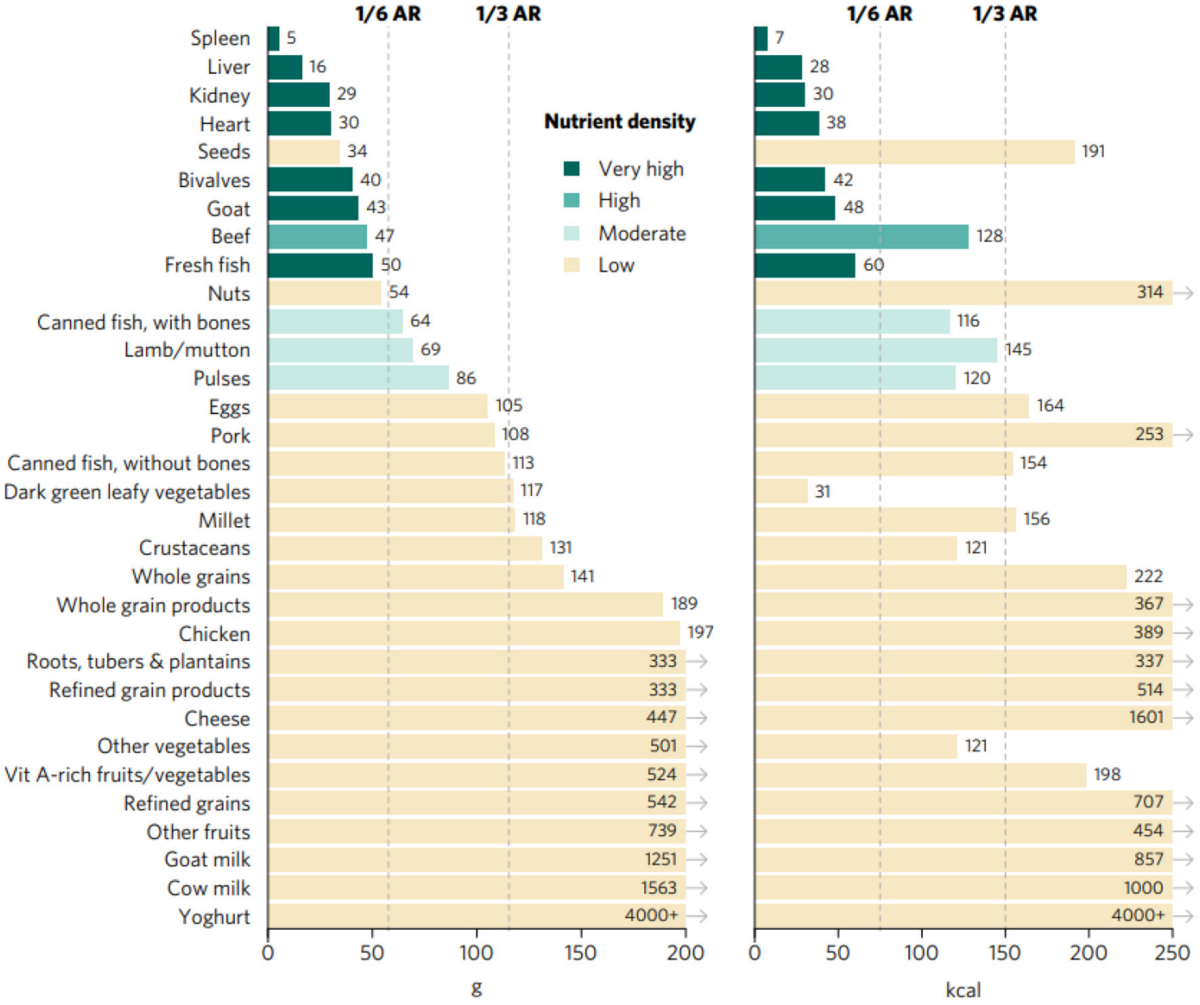
Portions sizes (calories and grams) needed to provide one-third of recommended iron intakes from complementary foods for children aged 6–23 months. Hypothetical average requirement (ARs) for mass were obtained by assuming an energy density of 1.3 kcal/g.

Top sources of iron included organs, fresh fish, bivalves, and goat, closely followed by beef and, to a lower extent, canned fish with bones, lamb/mutton, and pulses (Figures 2, 3). Organs, bivalves, fresh fish, and goat are also top zinc sources, together with crustaceans, beef, lamb/mutton, and eggs, followed by cheese, pork, and seeds. In addition to liver, kidney, bivalves, eggs, goat milk, and cheese, top vitamin A sources included two plant-source foods groups: DGLVs and vitamin A-rich fruits and vegetables, followed closely by cow milk, yogurt, and canned fish with bones. Cheese was identified as the only top source of calcium, closely followed by DGLVs, canned fish with bones, cow milk, goat milk, and yogurt. Top folate sources included liver, kidney, pulses, and whole grain products, followed by eggs, DGLVs, and seeds. Finally, all animal-source foods scored very high in vitamin B_12_, except for chicken and goat milk.

## Discussion

The purpose of this study was to identify top inherent food sources of multiple and individual micronutrients commonly lacking in complementary feeding diets of children aged 6–23 months in South and Southeast Asia. All analyzed animal-source foods, except for canned fish without bones, goat milk, pork and chicken, presented very high (organs, bivalves, crustaceans, goat, eggs, fresh fish, and canned fish with bones) or high (beef, lamb/mutton, cow milk, yogurt, and cheese) aggregate priority micronutrient density; while, DGLVs were the only plant-source food to score high for the aggregate micronutrient density rating. Our findings are in alignment with UNICEF's and WHO's (1, 3, 34, 38) recommendations for complementary feeding, according to which infants and young children should be fed animal-source foods daily or as often as possible, given that primarily plant-based or vegan diets, even when carefully planned, “*cannot meet nutrient needs at this age unless nutrient supplements or fortified products are used*” (1). Consumption of ASFs remains very low among children aged 6–23 months in South and Southeast Asia (6, 10, 39) and has been found to be strongly associated with infant and young child growth and development (11, 40). Results from the quantitative analysis conducted by UNICEF in its latest Child Nutrition Report (10) show that only 24% of children 6–23 months of age in South Asia consume eggs, fish, and/or meat. In alignment with UNICEF's findings, other recent evidence on South and Southeast Asia highlights that the vast majority of children aged 6–23 months are traditionally fed cereal- and pulse-based complementary foods for the most part (e.g., rice flour and rice porridge), while very few receive meat-, fish-, and/or egg-based complementary foods (2, 6, 9).

Unavailability and unaffordability, as well as insufficient maternal education, were identified as some of the main factors limiting consumption of animal-source foods and nutrient-dense foods in general among infants and young children in the two regions (2, 10, 41, 42). Indeed, while some foods of animal origin, such as liver, eggs and dairy, may be more available and affordable than others (particularly in terms of cost per nutrients provided), efforts to increase access to and knowledge around ASFs in South and Southeast Asia are still needed (43). Although this study does not aim (or claim) to provide solutions to the complex, multifaceted issues of availability, affordability, and desirability of foods, and of complementary feeding practices, it could make a positive contribution by helping policy-makers and program managers to identify the top food sources to prioritize when addressing micronutrient malnutrition in children aged 6–23 months. Policy-makers and program managers could use this knowledge to design and implement policies, measures, and programs toward increasing the availability and affordability of particularly nutrient-dense foods and improving complementary feeding practices among mothers and caregivers. Just to provide a few examples, among others they could (i) incentivize in-country/local production of these foods (e.g., through agricultural subsidies) to decrease the country's reliance on imports; (ii) increase investments in infrastructure services to reduce trade and transportation margins; (iii) provide targeted cash transfers to low-income households with children aged 6–23 months for the purchase of specific nutrient-dense foods; (iv) establish dedicated educational channels within public health systems and set up Social and Behavior Change Communication campaigns (e.g., through mass media and community mobilization) targeting mothers and caregivers of infants and young children (44).

Despite DGLVs being the only plant-source food with high aggregate priority micronutrient density, other PSFs scored very high or high in individual micronutrients. These PSFs could make an important contribution to the reduction of specific micronutrient gaps in complementary feeding diets, especially when appropriate low-cost, easy-to-implement processing techniques are adopted at the household level to increase absorption of non-heme iron and zinc, by reducing the negative effects of phytate on absorption [e.g., consumption of vitamin C, consumption of animal protein, heating, germination, soaking, and fermentation; (14)]. For instance, vitamin A deficiency in early childhood is widespread in South and Southeast Asia, and can have severe health consequences, which has led to countries' governments carrying out vitamin A supplementation campaigns targeted at infants and young children (6, 8). Consumption of micronutrient-dense fruits and vegetables, particularly DGLVs and vitamin A-rich fruits and vegetables (both scoring very high in vitamin A in our analysis) is very limited during the complementary feeding period in the two regions (2, 6). For instance, in South Asia only one in three (~33%) children aged 6–23 months receive these foods (2). Therefore, in addition to supplementation and fortification—both key strategies in the fight against micronutrient malnutrition—improving consumption of available, affordable vitamin A food sources in infants and young children would significantly contribute to the reduction of vitamin A deficiency in South and Southeast Asia (6, 43). Other examples of widely available, affordable plant-source foods with very high or high individual micronutrient density include: pulses and whole grain products for folate, and seeds for zinc and folate (in addition to DGLVs) (6, 38, 43).

Among the foods which scored low in all six included micronutrients, some, such as nuts, “other fruits” and “other vegetables” (non-vitamin A-rich and non-DGLVs), are often promoted as nutrient-dense in nutrition policies and programs to improve infants' and young children's diets in South and Southeast Asia and globally (6, 38). However, given the limited gastric capacity of infants and young children (1, 2), these foods could displace more nutrient-dense foods and prevent complementary fed children from obtaining adequate micronutrients necessary for proper growth and development. Though not particularly dense in priority micronutrients, moderate quantities of these foods, which provide energy and other essential nutrients, as well as non-essential beneficial compounds, can contribute to the overall quality and diversity of complementary feeding diets in South and Southeast Asia (2, 7, 38, 39).

This study has several strengths. First, while the importance of nutrient-dense foods, particularly ASFs, for infants and young children has already been extensively acknowledged in previous studies and guidelines (1, 3, 10, 34, 38, 45), this analysis brings added value to the literature by transparently ranking a diverse set of inherent food sources of two or more micronutrients commonly lacking during the complementary feeding period in South and Southeast Asia, and whose deprivation is the cause of significant public health burdens in these regions (6–9). It does so by developing a resource-inexpensive, reproducible approach, which is widely applicable in the two regions considered and could be easily adapted for use in other geographic areas of the world. Second, the approach used for rating foods takes into consideration infants' and young children's limited gastric capacity and plausible amounts of food they could consume at each meal and daily, as well as the adequate meal frequency during the complementary feeding period (1, 34). Third, the micronutrient density analysis was conducted based on an aggregate regional food composition database, which compiles data from multiple countries' FCTs and is reflective of the nutritional value of foods in Southern and Southeastern Asia, unlike other nutrient scoring systems which typically analyze data from a single national FCT, usually USDA FDC (46, 47). Fourth, iron and zinc values were adjusted for bioavailability in different foods. Finally, the methodology adopted is fully transparent, and the analysis is based on publicly available data, as has been recommended for nutrient profiling systems (46, 47). In addition, the results are presented both in written text and figures in a form that is easily interpretable by non-technical audiences, including policy makers and program managers.

The specific focus of this study on priority micronutrients is both a strength and a limitation. Indeed, in addition to the six micronutrients included in the analysis, foods provide energy, protein, essential amino acids, and fatty acids, as well as other essential vitamins and minerals, which are crucial to optimal growth and development in the first 2 years of a child's life and which can also be lacking to some extent in complementary feeding diets (1, 4, 9, 11, 22, 38, 48, 49). Moreover, minimally processed foods of both plant and animal origin contain countless non-essential compounds, including fiber, phytonutrients, and bioactive compounds, with potential beneficial effects on human health (50–53). However, this study focuses on micronutrients that are known to be commonly lacking in the two regions of interest and globally among infants and young children and are hindering optimal growth and development (7–9).

Other limitations should be acknowledged. First, the choice of countries' FCTs used and foods included in the regional food composition database was constrained by limitations in data quality and availability. Indeed, only one country FCT from South Asia (Bangladesh) was considered appropriate for inclusion, while all other selected FCTs are from Southeast Asia. Moreover, soy foods, as well as wild or indigenous fruits, vegetables, nuts, seeds, grains and pulses, many of which are more nutrient-dense than commercial varieties (54), have not been explored. Second, the adopted approach accounts for bioavailability of iron and zinc based on the heme-iron and phytate contents of foods, respectively, which are only two out of numerous factors influencing absorption, particularly the differing micronutrient status, overall diet and genetics of individuals. Third, there can be significant variations in nutrient values of a given food across countries' FCTs, which may be due to different varieties, soil characteristics, climate conditions, quantity and type of fertilizers used, animal feed, production, and processing methods, including local culinary traditions, as well as the quality of sampling, analysis, and reporting processes. However, this study attempts to mitigate such differences and uncertainties by building a regional food composition database with aggregate nutrient values from multiple national FCTs, including the robust USDA FDC. Fourth, as mentioned in the *Results* section, some of the analyzed food groups show high nutrient density variance across included foods, meaning that the overall score of an aggregate food group might not reflect the micronutrient density of all individual foods included. In this regard, certain foods (e.g., fruits and vegetables) may be more likely to be targeted in policies and programming as food groups rather than individually, and consequently they were aggregated despite presenting significant intra-group nutrient density variance.

In conclusion, results from this study clearly show that the introduction of small quantities of priority micronutrient-dense animal—(e.g., organs, fish and shellfish, eggs) and plant—(DGLVs) source foods would significantly contribute to achieving adequacy of micronutrients commonly lacking in complementary feeding diets in South and Southeast Asia. Noticeably, top sources of priority micronutrients should be consumed together with a variety of other nutrient-dense foods, as part of a diverse and balanced diet, able to meet all nutrient requirements of children aged 6–23 months. Our findings could be used to improve current countries' and regional recommendations on complementary feeding, by providing additional insights compared to just common knowledge on the high nutrient-density of ASFs and DGLV. Indeed, this study highlights the nutrient density of specific ASF (e.g., organ meats other than liver—spleen, kidney, heart—, bivalves, canned fish with bones) which are often not included in existing recommendations and whose potential remains largely unexplored. Also, our results show the differences in nutrient density among various ASFs and their relative ranking, enabling policy makers and program managers to prioritize certain ASFs over others for children 6–23 months. For instance, pork and chicken have lower priority micronutrient density compared to organs, ruminant meat, bivalves, eggs, cow milk, and others; therefore, the latter are more ideal types of ASFs to promote for feeding infants and young children on a regular basis. Further analyses are needed to explore ways to integrate these findings into food, agriculture, and nutrition policies and programs aiming to reduce micronutrient malnutrition in the first 2 years of life through the promotion of inherently nutrient-dense foods.

This study focuses specifically on infants and young children living in South and Southeast Asia, however the same approach could be used to analyze the priority micronutrient density of foods for complementary feeding in other regions of the world presenting similar micronutrient gaps in complementary feeding diets, such as Eastern and Southern Africa (55). Future research could build on this analysis, for instance, by expanding the regional food composition database through the inclusion of soy foods and of nutrient-dense wild or indigenous fruits, vegetables, nuts, seeds, grains, pulses (54), and insects (56), the safety and nutritional adequacy of which is currently being studied for potential application in complementary foods (57). In addition, findings from this study could be compared and complemented with affordability and environmental impact metrics, to assess these variables based on priority micronutrient density by expanding on existing approaches (13, 43, 58).

## Data Availability Statement

The original contributions presented in the study are included in the article/Supplementary Material, further inquiries can be directed to the corresponding author.

## Author Contributions

FO and TB designed the study and conducted the analyses. Both authors contributed to the article and approved the submitted version.

## Funding

This work was funded by contributions from the Ministry of the Foreign Affairs of the Netherlands (grant #4000000622 to GAIN) and the Bill & Melinda Gates Foundation through the Regional Initiatives for Sustained Improvements in Nutrition and Growth (grant INV-008600 to UNICEF). The funder had no role in data collection and analysis, manuscript preparation and revision, or the decision to publish. This study used data from public sources, and all authors had access to the data analyzed as part of this study.

## Author Disclaimer

The findings and conclusions contained within are those of the authors and do not necessarily reflect positions or policies of the funder.

## Conflict of Interest

The authors declare that the research was conducted in the absence of any commercial or financial relationships that could be construed as a potential conflict of interest.

## Publisher's Note

All claims expressed in this article are solely those of the authors and do not necessarily represent those of their affiliated organizations, or those of the publisher, the editors and the reviewers. Any product that may be evaluated in this article, or claim that may be made by its manufacturer, is not guaranteed or endorsed by the publisher.

## Footnotes

^1^Fortified foods not included.

^2^In particular, vitamin B_12_ from the Bangladesh FCT.

^3^Obtained by averaging the energy densities of a minimally processed plant-based, low-fat diet and a minimally processed, animal-based, ketogenic diet (31).
