# Supplementary material for: Priority Micronutrient Density of Foods for Complementary Feeding of Young Children (6–23 Months) in South and Southeast Asia
Source: Front Nutr. 2021 Dec 21;8:785227. doi: 10.3389/fnut.2021.785227 (PMC8724761; doi:10.3389/fnut.2021.785227)
Supplement: Supplementary file 1 [file Data_Sheet_1.docx]

***Supplementary Material***

Table of Contents

[Supplemental methods section 2](#_Toc88517832)

[Calculating Recommended Nutrient Intakes 2](#_Toc88517833)

[Rationale for excluding fortified foods 2](#_Toc88517834)

[Selection criteria for countries’ food composition tables 3](#_Toc88517835)

[Accounting for iron bioavailability of foods 3](#_Toc88517836)

[Accounting for zinc bioavailability of foods 3](#_Toc88517837)

[Supplemental Table 1: First level granular food composition database for plant-source foods (PSFs) 6](#_Toc88517888)

[Supplemental Table 2: Second level granular food composition database for plant-source foods (PSFs) 8](#_Toc88517889)

[Supplemental Table 3: First level granular food composition data on animal-source foods (ASFs) 15](#_Toc88517890)

[Supplemental Table 4: Second level granular food composition database on animal-source foods (ASFs) 18](#_Toc88517891)

# Supplemental methods section

## Calculating R**ecommended Nutrient Intakes**

We started from calculating total Recommended Nutrient Intakes (RNIs), to be achieved through the combination of breast milk (or formula) and complementary foods, for children aged 6-23 months. Following a previously adopted approach to identify affordable nutritious complementary foods (1,2), total RNIs for the age group of our interest (6-23 months):

- for calcium, zinc and iron were obtained by calculating the weighted average of the World Health Organization (WHO) and the Food and Agriculture Organization (FAO) recommendations (3) for children aged 6-11 months and 12-35 months;
- for folate, vitamin A and vitamin B_12_ were derived by calculating the weighted average of the Institute of Medicine (IOM) recommendations (4) for children aged 6-11 months and 12-35 months.

The next step consisted in calculating the proportion of RNIs to be achieved through complementary foods, assuming that the remaining proportion would be provided by breast milk (or formula) (5) and considering the average breast milk intake in developing countries (6,7).

Moreover, RNIs for iron and zinc were adjusted for bioavailability. Three levels of iron absorption (20%, 15%, 10%) and the corresponding recommended iron intakes were considered in the analysis (8,9). For zinc, four levels of absorption (44%, 35%, 30%, 26%) were considered, based on dietary phytate intake: the higher the phytate intake, the lower the zinc absorption level, the higher the recommended zinc intake (9,10).

Finally, Average Energy Requirements (AERs) from complementary foods for children aged 6-23 months were obtained by calculating the weighted average of the WHO and the United Nations Children’s Fund (UNICEF) recommendations (6,7) for children aged 6-8 months, 9-11 months and 12-23 months, accounting for average breast milk intake in developing countries.

## Rationale for excluding fortified foods

Although we fully recognize the crucial importance of food fortification as an efficient and cost-effective strategy to reduce micronutrient malnutrition in low- and middle-income countries (LMICs) (11), fortified staple foods (e.g. wheat flour, white rice) cannot be considered equal to and directly comparable with minimally processed, inherently nutrient-dense foods, particularly in terms of health effects. The reason for this is that there are more than 70,000 compounds in foods (12) bound together in a complex food matrix, which act synergistically on metabolism, including nutrient absorption, and on which there is increasing evidence for potential positive health implications (13–16). Therefore, achieving micronutrient adequacy from minimally processed foods may have additional benefits beyond fortification due to the added value of the food matrix (13–16), which is our rationale for focusing on inherently nutrient-dense food sources.

## Selection criteria for countries’ food composition tables

Available countries’ food composition tables (FCTs) were not included in the compiled regional food composition database for South and Southeast Asia if one or more of the following conditions were verified: (a) they did not include values for foods in the forms typically consumed (e.g. many FCTs *only* included raw foods); (b) they borrowed values for several foods from other tables (usually USDA FoodData Central, FDC (17)) rather than reporting their own original values; (c) they presented numerous suspect values when comparing them with USDA FDC, used as a reference for plausibility and accuracy of data; (d) they did not include values for all six priority micronutrients.

## Accounting for iron bioavailability of foods

Assuming 10% and 25% bioavailability for non-heme iron and heme iron, respectively (8), the following calculations were executed:

- Ruminant meat (including beef, goat, lamb/mutton):
  - Heme iron content: 68%
  - Calculated iron bioavailability: 68% * 0.25 + 32% * 0.10 = 20%
- Pork:
  - Heme iron content: 39%
  - Calculated iron bioavailability: 39% * 0.25 + 61% * 0.10 = 16%
- Chicken, fish and seafood, eggs and dairy:
  - Heme iron content: 26%
  - Calculated iron bioavailability: 26% * 0.25 + 74% * 0.10 = 14%
- All other meat, including offal:
  - Heme iron content: 40%
  - Calculated iron bioavailability: 40% * 0.25 + 60% * 0.10 = 16%
- All plant-source foods:
  - Heme iron content: 0%
  - Calculated iron bioavailability: 0% * 0.25 + 100% * 0.10 = 10%

Based on the above calculations, foods were assigned to one of three levels of iron absorption: 20% for ruminant meat, 15% for all other animal-source foods (ASFs), and 10% for all plant-source foods (PSFs), following an approach previously used by the authors to conduct other micronutrient density analyses (9).

## Accounting for zinc bioavailability of foods

Foods were categorized into four levels of zinc absorption (44%, 35%, 30% and 26%), based on the amount of phytate contained in a portion equivalent to one-third of daily mass intake, assuming an energy density of 1.3 kcal/g and considering average energy requirements for women of reproductive age (10). In particular, the lower limits of thresholds on daily phytate intake identified by European Food Safety Authority (EFSA) (10) were used. Such thresholds apply to the phytate content of diets as a whole rather than of individual foods. Indeed, any food is consumed within a broader diet and foods constituting the hypothetical remaining two-thirds of the diet will likely contribute additional phytate. However, the lower limits of EFSA’s thresholds on daily phytate intake were chosen so that the four zinc absorption categories would reflect bioavailability when attempting to moderate overall dietary phytate, because of its anti-nutrient effect on mineral absorption. Thus, the four levels for zinc absorption were based on the following phytate ranges in the selected food:

- Refined diet: ≤ 300 mg
- Semi-refined: 301–600 mg
- Semi-unrefined: 601–900 mg
- Unrefined: > 900 mg

The higher the phytate intake range, the lower the zinc absorption level. For instance, the refined diet category presents the lowest phytate intake range and the highest zinc absorption level, while the unrefined diet category is the exact opposite. The semi-refined and semi-unrefined diet categories are somewhere in between, with intermediate phytate intake ranges and intermediate zinc absorption levels. The same approach has been previously used by the authors to conduct other micronutrient density analyses (9).

**References**

1. Beal T, White JM, Arsenault JE, Okronipa H, Hinnouho G-M, Torlesse H, et al. Micronutrient gaps during the complementary feeding period in South Asia: A Comprehensive Nutrient Gap Assessment. Nutrition Reviews. 2021;79(Suppl 4).

2. Ryckman T, Beal T, Nordhagen S, Chimanya K, Matji J. Affordability of nutritious foods for complementary feeding in Eastern and Southern Africa. Nutrition Reviews. 2021;79(4).

3. World Health Organization, Food and Agriculture Organization of the United Nations (FAO). Vitamin and mineral requirements in human nutrition [Internet]. 2nd ed. 2005 [cited 2021 Aug 17]. 341 p. Available from: https://apps.who.int/iris/handle/10665/42716

4. Institute of Medicine Committee to Review Dietary Reference Intakes for Vitamin D and Calcium. Dietary Reference Intakes for Calcium and Vitamin D. Ross AC, Taylor CL, Yaktine AL, Del Valle HB, editors. Washington, DC: National Academies Press; 2011. (The National Academies Collection: Reports funded by National Institutes of Health).

5. Dewey KG. Nutrition, Growth, and Complementary Feeding of The Brestfed Infant. Pediatric Clinics of North America. 2001 Feb 1;48(1):87–104.

6. Pan American Health Organization. Guiding principles for complementary feeding of the breastfed child [Internet]. 2003 [cited 2021 Aug 16]. Available from: https://www.who.int/nutrition/publications/guiding_principles_compfeeding_breastfed.pdf

7. WHO Programme of Nutrition. Complementary feeding of young children in developing countries : a review of current scientific knowledge [Internet]. World Health Organization; 1998 [cited 2021 Aug 16]. Report No.: WHO/NUT/98.1. Available from: https://apps.who.int/iris/handle/10665/65932

8. Beal T, Massiot E, Arsenault JE, Smith MR, Hijmans RJ. Global trends in dietary micronutrient supplies and estimated prevalence of inadequate intakes. PLOS ONE. 2017 Apr 11;12(4):e0175554.

9. Beal T, Ortenzi F. Priority micronutrient density in foods [Internet]. 2021 [cited 2021 Aug 17]. Available from: https://www.researchsquare.com/article/rs-701840/v1

10. European Food Safety Authority (EFSA). Dietary Reference Values for nutrients Summary report [Internet]. 2017 [cited 2021 Aug 18]. (EFSA Supporting Publications). Report No.: 14. Available from: https://www.efsa.europa.eu/en/supporting/pub/e15121

11. Osendarp SJM, Martinez H, Garrett GS, Neufeld LM, De-Regil LM, Vossenaar M, et al. Large-Scale Food Fortification and Biofortification in Low- and Middle-Income Countries: A Review of Programs, Trends, Challenges, and Evidence Gaps. Food Nutr Bull. 2018 Jun 1;39(2):315–31.

12. Listing Compounds - FooDB [Internet]. [cited 2021 Nov 22]. Available from: https://foodb.ca/compounds

13. van Vliet S, Kronberg SL, Provenza FD. Plant-Based Meats, Human Health, and Climate Change. Frontiers in Sustainable Food Systems. 2020;4:128.

14. Barabási A-L, Menichetti G, Loscalzo J. The unmapped chemical complexity of our diet. Nat Food. 2020 Jan;1(1):33–7.

15. Jacobs DR, Tapsell LC. Food, Not Nutrients, Is the Fundamental Unit in Nutrition. Nutrition Reviews. 2007 Oct 1;65(10):439–50.

16. Aguilera JM. The food matrix: implications in processing, nutrition and health. Critical Reviews in Food Science and Nutrition. 2019 Dec 16;59(22):3612–29.

17. U.S. DEPARTMENT OF AGRICULTURE (USDA), Agricultural Research Service. USDA FoodData Central [Internet]. [cited 2021 Jun 18]. Available from: https://fdc.nal.usda.gov/

Supplemental Table 1: First level granular food composition database for plant-source foods (PSFs)

| **Food Group** | **Food (100 g)** | **Country FCT or regional composite FCT** | **kcal** | **Vit A (mcg RAE)** | **Folate (mcg DFE)** | **Vit B_12_ (mcg)** | **Calcium (mg)** | **Iron (mg)** | **Zinc (mg)** | **Phytate (mg)** | **Comments** |
| --- | --- | --- | --- | --- | --- | --- | --- | --- | --- | --- | --- |
| **Pulses** | Pulses | USDA | 121.50 | 0.50 | 157.50 | 0.00 | 26.00 | 2.92 | 1.28 | 404.56 |  |
|  | Pulses | South/Southeast Asia | 158.00 | 1.50 | 37.00 | 0.00 | 27.00 | 2.55 | 1.25 | 476.48 |  |
|  | Pulses | **Composite** | 139.75 | 1.00 | 97.25 | 0.00 | 26.50 | 2.74 | 1.26 | 440.52 |  |
| **Whole grains** | Whole grains | USDA | 122.00 | 0.00 | 16.00 | 0.00 | 3.00 | 0.56 | 0.71 | 437.58 |  |
|  | Whole grains | South/Southeast Asia | 193.26 | 0.00 | 14.94 | 0.00 | 23.03 | 2.75 | 1.49 | 448.80 | Only whole wheat from Bangladesh: no other whole grains available from any of the selected Asian FCTs |
|  | Whole grains | **Composite** | 157.63 | 0.00 | 15.47 | 0.00 | 13.02 | 1.66 | 1.10 | 443.19 |  |
| **Refined grains** | Refined grains | USDA | 130.00 | 0.00 | 6.00 | 0.00 | 11.00 | 0.46 | 0.69 | 47.47 |  |
|  | Refined grains | South/Southeast Asia | 131.00 | 0.00 | 4.00 | 0.00 | 7.00 | 0.40 | 0.60 | 35.76 |  |
|  | Refined grains | **Composite** | 130.50 | 0.00 | 5.00 | 0.00 | 9.00 | 0.43 | 0.65 | 41.62 |  |
| **Whole grain products** | Whole grain products | USDA | 194.00 | 0.00 | 84.00 | 0.00 | 27.50 | 1.24 | 1.15 | 77.00 | Values from USDA FDC only; no whole grain products available in any of the selected Asian FCTs |
| **Refined grain products** | Refined grain products | USDA | 158.00 | 0.00 | 7.00 | 0.00 | 7.00 | 0.50 | 0.51 | 49.26 |  |
|  | Refined grain products | South/Southeast Asia | 151.00 | 0.00 | 5.00 | 0.00 | 11.00 | 0.90 | 0.57 | 49.26 |  |
|  | Refined grain products | **Composite** | 154.50 | 0.00 | 6.00 | 0.00 | 9.00 | 0.70 | 0.54 | 49.26 |  |
| **Traditional grains** | Millet | USDA | 119.00 | 0.00 | 19.00 | 0.00 | 3.00 | 0.63 | 0.91 | 200.00 |  |
|  | Millet | South/Southeast Asia | 145.42 | 0.00 | 26.56 | 0.00 | 17.50 | 3.33 | 1.26 | 200.00 |  |
|  | Millet | **Composite** | 132.21 | 0.00 | 22.78 | 0.00 | 10.25 | 1.98 | 1.09 | 200.00 |  |
| **Nuts** | Nuts | USDA | 576.50 | 0.00 | 62.00 | 0.00 | 75.00 | 3.83 | 3.20 | 709.50 |  |
|  | Nuts | South/Southeast Asia | 585.00 | 1.00 | 98.00 | 0.00 | 76.00 | 4.80 | 3.09 | 582.00 |  |
|  | Nuts | **Composite** | 580.75 | 0.50 | 80.00 | 0.00 | 75.50 | 4.32 | 3.15 | 645.75 |  |
| **Seeds** | Seeds | USDA | 569.00 | 0.00 | 97.50 | 0.00 | 120.50 | 7.44 | 6.23 | 381.24 |  |
|  | Seeds | South/Southeast Asia | 552.00 | 1.00 | 97.00 | 0.00 | 147.00 | 6.40 | 5.00 | 423.00 |  |
|  | Seeds | **Composite** | 560.50 | 0.50 | 97.25 | 0.00 | 133.75 | 6.92 | 5.62 | 402.12 |  |
| **Starchy roots, tubers and plantains** | Starchy roots, tubers and plantains | USDA | 104.50 | 29.00 | 15.50 | 0.00 | 14.50 | 0.60 | 0.27 | 12.00 |  |
|  | Starchy roots, tubers and plantains | South/Southeast Asia | 98.00 | 3.00 | 7.00 | 0.00 | 29.00 | 0.80 | 0.32 | 12.00 |  |
|  | Starchy roots, tubers and plantains | **Composite** | 101.25 | 16.00 | 11.25 | 0.00 | 21.75 | 0.70 | 0.30 | 12.00 |  |
| **DGLVs** | DGLVs | USDA | 21.00 | 306.00 | 38.00 | 0.00 | 58.00 | 2.26 | 0.27 | 23.01 |  |
|  | DGLVs | South/Southeast Asia | 32.00 | 266.00 | 36.00 | 0.00 | 145.00 | 1.75 | 0.35 | 8.51 |  |
|  | DGLVs | **Composite** | 26.50 | 286.00 | 37.00 | 0.00 | 101.50 | 2.01 | 0.31 | 15.76 |  |
| **Vit A-rich fruits and veg, excl DGLVs** | Vit A-rich fruits and vegetables, excluding DGLVs | USDA | 40.50 | 147.50 | 20.00 | 0.00 | 13.50 | 0.39 | 0.18 | 22.50 |  |
|  | Vit A-rich fruits and vegetables, excluding DGLVs | South/Southeast Asia | 35.00 | 105.00 | 21.00 | 0.00 | 26.00 | 0.50 | 0.17 | 25.00 |  |
|  | Vit A-rich fruits and vegetables, excluding DGLVs | **Composite** | 37.75 | 126.25 | 20.50 | 0.00 | 19.75 | 0.45 | 0.18 | 23.75 |  |
| **Other fruits, excl vit A-rich fruits** | Other fruits, excluding vit A-rich fruits | USDA | 60.50 | 3.00 | 19.00 | 0.00 | 11.00 | 0.28 | 0.11 | 10.00 |  |
|  | Other fruits, excluding vit A-rich fruits | South/Southeast Asia | 62.50 | 5.00 | 15.00 | 0.00 | 11.50 | 0.35 | 0.09 | 10.00 |  |
|  | Other fruits, excluding vit A-rich fruits | **Composite** | 61.50 | 4.00 | 17.00 | 0.00 | 11.25 | 0.32 | 0.10 | 10.00 |  |
| **Other vegetables, excl DGLVs & vit A-rich veg** | Other vegetables, excluding DGLVs and vit A-rich vegetables | USDA | 22.50 | 20.50 | 15.50 | 0.00 | 13.50 | 0.33 | 0.17 | 13.00 |  |
|  | Other vegetables, excluding DGLVs and vit A-rich vegetables | South/Southeast Asia | 26.00 | 9.00 | 15.00 | 0.00 | 24.00 | 0.60 | 0.34 | 9.10 |  |
|  | Other vegetables, excluding DGLVs and vit A-rich vegetables | **Composite** | 24.25 | 14.75 | 15.25 | 0.00 | 18.75 | 0.47 | 0.26 | 11.05 |  |

Supplemental Table 2: Second level granular food composition database for plant-source foods (PSFs)

| **Country FCT** | **Food Group** | **Food (100 g)** | **kcal** | **Vit A (mcg RAE)** | **Folate (mcg DFE)** | **Vit B_12_ (mcg)** | **Calcium (mg)** | **Iron (mg)** | **Zinc (mg)** | **Phytate (mg)** | **Comments** |
| --- | --- | --- | --- | --- | --- | --- | --- | --- | --- | --- | --- |
| **USDA** | Pulses | Lentils, mature seeds, cooked, boiled, without salt | 116.00 | 0.00 | 181.00 | 0.00 | 19.00 | 3.33 | 1.27 | 316.40 |  |
|  |  | Chickpea, mature seeds, cooked, boiled, without salt | 164.00 | 1.00 | 172.00 | 0.00 | 49.00 | 2.89 | 1.53 | 566.35 |  |
|  |  | Cowpea, common, mature seeds, cooked, boiled, without salt | 116.00 | 1.00 | 143.00 | 0.00 | 24.00 | 2.51 | 1.29 | 492.72 |  |
|  |  | Beans, kidney, red, mature seeds, cooked, boiled, with salt | 127.00 | 0.00 | 130.00 | 0.00 | 28.00 | 2.94 | 1.07 | 219.00 |  |
|  |  | **USDA composite** | 121.50 | 0.50 | 157.50 | 0.00 | 26.00 | 2.92 | 1.28 | 404.56 |  |
| **Bangladesh** | Pulses | Bengal gram, whole, boiled* (without salt) | 182.00 | 2.00 | 48.00 | 0.00 | 94.00 | 4.00 | 1.26 | 566.35 |  |
|  |  | Green gram, split, boiled* (without salt) | 161.00 | 1.00 | 32.00 | 0.00 | 30.00 | 2.90 | 1.13 | 476.48 |  |
|  |  | Grass pea, split, boiled* (without salt) | 142.00 | 2.00 | 42.00 | 0.00 | 24.00 | 1.90 | 1.23 |  | Phytate value unavailable |
|  |  | Lentils, boiled* (without salt) | 155.00 | 1.00 | 9.00 | 0.00 | 12.00 | 2.20 | 1.72 | 316.40 |  |
|  |  | **South/Southeast Asia composite** | 158.00 | 1.50 | 37.00 | 0.00 | 27.00 | 2.55 | 1.25 | 476.48 |  |
| **USDA** | Whole grains | Wheat, KAMUT khorasan, cooked | 132.00 | 4.00 | 11.00 | 0.00 | 9.00 | 1.76 | 1.84 | 448.80 |  |
|  |  | Corn, sweet, white, cooked, boiled, drained, without salt | 97.00 | 0.00 | 20.00 | 0.00 | 2.00 | 0.55 | 0.54 | 426.36 |  |
|  |  | Corn, sweet, yellow, cooked, boiled, drained, without salt | 96.00 | 13.00 | 23.00 | 0.00 | 3.00 | 0.45 | 0.62 | 426.36 |  |
|  |  | Rice, brown, cooked, no fat added | 122.00 | 0.00 | 9.00 | 0.00 | 3.00 | 0.56 | 0.71 | 612.16 |  |
|  |  | Barley, cooked, no added fat | 122.00 | 0.00 | 16.00 | 0.00 | 11.00 | 1.32 | 0.82 |  | Phytate value unavailable |
|  |  | **USDA composite** | 122.00 | 0.00 | 16.00 | 0.00 | 3.00 | 0.56 | 0.71 | 437.58 |  |
| **Bangladesh** | Whole grains | Wheat, whole, boiled | 193.26 | 0.00 | 14.94 | 0.00 | 23.03 | 2.75 | 1.49 | 448.80 | Used weight yields and nutrient retention factors |
| **USDA** | Refined grains | Rice, white, medium-grain, cooked, unenriched | 130.00 | 0.00 | 2.00 | 0.00 | 3.00 | 0.20 | 0.42 | 47.47 |  |
|  |  | Rice, fried, meatless | 174.00 | 22.00 | 6.00 | 0.00 | 11.00 | 0.46 | 0.69 |  | Phytate value unavailable |
|  |  | Barley, pearled, cooked | 123.00 | 0.00 | 16.00 | 0.00 | 11.00 | 1.33 | 0.82 |  | Phytate value unavailable |
|  |  | **USDA composite** | 130.00 | 0.00 | 6.00 | 0.00 | 11.00 | 0.46 | 0.69 | 47.47 |  |
| **Bangladesh** | Refined grains | Rice, white, sunned, aromatic, boiled* (without salt) | 110.00 | 0.00 | 2.00 | 0.00 | 6.00 | 0.40 | 0.33 | 47.57 |  |
| **Laos** | Refined grains | Rice, steamed, white | 131.00 | 0.00 | 4.00 | 0.00 | 7.00 | 0.40 | 0.60 | 23.94 |  |
|  |  | Rice, white, fried | 370.00 | 0.00 | 7.00 | 0.00 | 11.00 | 1.60 | 1.20 |  | Phytate value unavailable |
|  |  | **South/Southeast Asia composite** | 131.00 | 0.00 | 4.00 | 0.00 | 7.00 | 0.40 | 0.60 | 35.76 |  |
| **USDA** | Whole grain products | Bread, french or vienna, whole wheat | 239.00 | 0.00 | 131.00 | 0.00 | 42.00 | 0.75 | 1.16 | 49.00 |  |
|  |  | Bread, rye | 259.00 | 0.00 | 151.00 | 0.00 | 73.00 | 2.83 | 1.14 | 77.00 |  |
|  |  | Pasta, whole wheat, cooked | 149.00 | 0.00 | 21.00 | 0.00 | 13.00 | 1.72 | 1.34 |  | Phytate value unavailable |
|  |  | Cornmeal mush, no added fat | 65.00 | 2.00 | 37.00 | 0.00 | 4.00 | 0.65 | 0.11 | 198.98 |  |
|  |  | **USDA composite** | 194.00 | 0.00 | 84.00 | 0.00 | 27.50 | 1.24 | 1.15 | 77.00 |  |
| **USDA** | Refined grain products | Bread, pita, white, unenriched | 275.00 | 0.00 | 24.00 | 0.00 | 86.00 | 1.40 | 0.84 | 49.26 |  |
|  |  | Pasta, cooked, unenriched, WO added salt | 158.00 | 0.00 | 7.00 | 0.00 | 7.00 | 0.50 | 0.51 |  | Phytate value unavailable |
|  |  | Rice noodles, cooked | 108.00 | 0.00 | 1.00 | 0.00 | 4.00 | 0.14 | 0.25 |  | Phytate value unavailable |
|  |  | **USDA composite** | 158.00 | 0.00 | 7.00 | 0.00 | 7.00 | 0.50 | 0.51 | 49.26 |  |
| **Bangladesh** | Refined grain products | Vermicelli, wheat, boiled, WO salt | 151.00 | 0.00 | 5.00 | 0.00 | 11.00 | 0.90 | 0.57 | 164.74 |  |
| **Vietnam** | Refined grain products | Bread, French style | 249.00 | 0.00 | 33.00 | 0.00 | 28.00 | 2.00 | 0.85 | 49.26 |  |
| **Laos** | Refined grain products | Porridge, white rice, boiled | 59.00 | 0.00 | 1.00 | 0.00 | 2.00 | 0.13 | 0.19 | 20.00 |  |
|  |  | **South/Southeast Asia composite** | 151.00 | 0.00 | 5.00 | 0.00 | 11.00 | 0.90 | 0.57 | 49.26 |  |
| **USDA** | Traditional grains | Millet, cooked | 119.00 | 0.00 | 19.00 | 0.00 | 3.00 | 0.63 | 0.91 | 200.00 |  |
| **Bangladesh** | Traditional grains | Pearl millet, whole-grain, stewed | 145.42 | 0.00 | 28.33 | 0.00 | 17.50 | 3.33 | 1.29 | 200.00 | Used weight yields and nutrient retention factors |
|  |  | Pearl millet, whole-grain, boiled | 145.42 | 0.00 | 24.79 | 0.00 | 17.50 | 3.33 | 1.23 | 200.00 | Used weight yields and nutrient retention factors |
|  |  | **South/Southeast Asia composite** | 145.42 | 0.00 | 26.56 | 0.00 | 17.50 | 3.33 | 1.26 | 200.00 |  |
| **USDA** | Nuts | Peanuts, all types, dry-roasted, WO salt | 587.00 | 0.00 | 97.00 | 0.00 | 58.00 | 1.58 | 2.77 | 2008.00 |  |
|  |  | Peanuts, all types, raw | 567.00 | 0.00 | 240.00 | 0.00 | 92.00 | 4.58 | 3.27 | 582.00 |  |
|  |  | Nuts, cashew nuts, raw | 553.00 | 0.00 | 25.00 | 0.00 | 37.00 | 6.68 | 5.78 | 290.00 |  |
|  |  | Nuts, cashew nuts, dry roasted, without salt added | 574.00 | 0.00 | 69.00 | 0.00 | 45.00 | 6.00 | 5.60 | 1229.00 |  |
|  |  | Nuts, almonds | 579.00 | 0.00 | 44.00 | 0.00 | 269.00 | 3.71 | 3.12 | 964.00 |  |
|  |  | Nuts, almonds, dry roasted, without salt added | 598.00 | 0.00 | 55.00 | 0.00 | 268.00 | 3.73 | 3.31 | 2111.00 |  |
|  |  | Nuts, pistachio nuts, raw | 560.00 | 26.00 | 51.00 | 0.00 | 105.00 | 3.92 | 2.20 | 808.00 |  |
|  |  | Nuts, walnuts | 654.00 | 1.00 | 98.00 | 0.00 | 98.00 | 2.91 | 3.09 | 611.00 |  |
|  |  | Peanuts, all types, cooked, boiled, with salt | 318.00 | 0.00 | 75.00 | 0.00 | 55.00 | 1.01 | 1.83 | 505.00 |  |
|  |  | Nuts, pine nuts, dried | 673.00 | 1.00 | 34.00 | 0.00 | 16.00 | 5.53 | 6.45 | 200.00 |  |
|  |  | **USDA composite** | 576.50 | 0.00 | 62.00 | 0.00 | 75.00 | 3.83 | 3.20 | 709.50 |  |
| **Bangladesh** | Nuts | Cashew nuts, raw | 595.00 | 1.00 | 25.00 | 0.00 | 50.00 | 5.00 | 5.78 | 290.00 |  |
|  |  | Groundnuts/peanut, raw | 585.00 | 0.00 | 175.00 | 0.00 | 76.00 | 2.90 | 3.39 | 582.00 |  |
|  |  | Pistachio nuts, dried | 574.00 | 16.00 | 51.00 | 0.00 | 117.00 | 5.30 | 2.20 | 808.00 |  |
|  |  | Walnuts | 684.00 | 1.00 | 98.00 | 0.00 | 100.00 | 4.80 | 3.09 | 611.00 |  |
| **Vietnam** | Nuts | Peanut, dried | 573.00 | 1.00 | 240.00 | 0.00 | 68.00 | 2.20 | 1.90 | 582.00 |  |
|  |  | **South/Southeast Asia composite** | 585.00 | 1.00 | 98.00 | 0.00 | 76.00 | 4.80 | 3.09 | 582.00 |  |
| **USDA** | Seeds | Seeds, pumpkin and squash seed kernels, dried | 559.00 | 1.00 | 58.00 | 0.00 | 46.00 | 8.82 | 7.81 | 24.25 |  |
|  |  | Seeds, pumpkin and squash seed kernels, roasted, without salt | 574.00 | 0.00 | 57.00 | 0.00 | 52.00 | 8.07 | 7.64 | 56.10 |  |
|  |  | Seeds, flaxseed | 534.00 | 0.00 | 87.00 | 0.00 | 255.00 | 5.73 | 4.34 | 1859.00 |  |
|  |  | Seeds, sesame seeds, whole, dried | 573.00 | 0.00 | 97.00 | 0.00 | 975.00 | 14.60 | 7.75 | 1525.00 |  |
|  |  | Seeds, sesame seeds, whole, roasted and toasted | 565.00 | 0.00 | 98.00 | 0.00 | 989.00 | 14.80 | 7.16 |  | Phytate value unavailable |
|  |  | Seeds, sunflower seed kernels, dried | 584.00 | 3.00 | 227.00 | 0.00 | 78.00 | 5.25 | 5.00 | 423.00 |  |
|  |  | Seeds, sunflower seed kernels, toasted, without salt | 619.00 | 0.00 | 238.00 | 0.00 | 57.00 | 6.81 | 5.30 |  | Phytate value unavailable |
|  |  | Seeds, lotus seeds, dried | 332.00 | 3.00 | 104.00 | 0.00 | 163.00 | 3.53 | 1.05 | 339.48 |  |
|  |  | **USDA composite** | 569.00 | 0.00 | 97.50 | 0.00 | 120.50 | 7.44 | 6.23 | 381.24 |  |
| **Bangladesh** | Seeds | Sunflower seeds, dried | 552.00 | 2.00 | 227.00 | 0.00 | 78.00 | 5.30 | 5.00 | 423.00 |  |
|  |  | Linseeds, tisi, raw | 500.00 | 0.00 | 87.00 | 0.00 | 255.00 | 5.70 | 4.34 | 1859.00 |  |
|  |  | Pumpkin seeds, dried | 580.00 | 1.00 | 58.00 | 0.00 | 45.00 | 8.10 | 7.21 | 24.25 |  |
|  |  | Sesame seeds, whole, dried | 563.00 | 0.00 | 97.00 | 0.00 | 969.00 | 10.50 | 7.70 | 1525.00 |  |
|  |  | Lotus seeds, dried | 339.00 | 3.00 | 104.00 | 0.00 | 147.00 | 5.60 | 1.05 | 339.48 |  |
| **Vietnam** | Seeds | Lotus seed, dried | 334.00 | 3.00 | 104.00 | 0.00 | 89.00 | 6.40 | 1.05 | 339.48 |  |
|  |  | Sesame seeds, dried | 568.00 | 1.00 | 97.00 | 0.00 | 975.00 | 14.60 | 7.75 | 1525.00 |  |
|  |  | **South/Southeast Asia composite** | 552.00 | 1.00 | 97.00 | 0.00 | 147.00 | 6.40 | 5.00 | 423.00 |  |
| **USDA** | Starchy roots, tubers and plantains | Potatoes, baked, flesh and skin, without salt | 93.00 | 1.00 | 28.00 | 0.00 | 15.00 | 1.08 | 0.36 |  | Phytate value unavailable |
|  |  | Potatoes, boiled, cooked in skin, flesh, without salt | 87.00 | 0.00 | 10.00 | 0.00 | 5.00 | 0.31 | 0.30 | 4.82 |  |
|  |  | Cassava, cooked | 191.00 | 13.00 | 24.00 | 0.00 | 17.00 | 0.28 | 0.36 | 60.00 |  |
|  |  | Sweet potato, cooked, baked in skin, flesh, without salt | 90.00 | 961.00 | 6.00 | 0.00 | 38.00 | 0.69 | 0.32 |  | Phytate value unavailable |
|  |  | Sweet potato, boiled, no added fat | 76.00 | 784.00 | 6.00 | 0.00 | 27.00 | 0.72 | 0.20 | 12.00 |  |
|  |  | Yam, cooked, boiled, drained, or baked, without salt | 116.00 | 6.00 | 16.00 | 0.00 | 14.00 | 0.52 | 0.20 | 50.00 |  |
|  |  | Plantains, green, fried | 309.00 | 60.00 | 15.00 | 0.00 | 4.00 | 0.67 | 0.23 | 0.57 |  |
|  |  | Plantains, yellow, baked | 155.00 | 45.00 | 53.00 | 0.00 | 3.00 | 0.28 | 0.21 |  | Phytate value unavailable |
|  |  | **USDA composite** | 104.50 | 29.00 | 15.50 | 0.00 | 14.50 | 0.60 | 0.27 | 12.00 |  |
| **Bangladesh** | Starchy roots, tubers and plantains | Colocasia/Taro, boiled* (without salt) | 115.00 | 4.00 | 16.00 | 0.00 | 40.00 | 0.80 | 0.23 | 18.10 |  |
|  |  | Potato, Diamond, boiled* (without salt) | 67.00 | 2.00 | 11.00 | 0.00 | 14.00 | 0.60 | 0.72 | 4.82 |  |
|  |  | Sweet potato, Komola Sundori, orange flesh, boiled* (without salt) | 98.00 | 692.00 | 7.00 | 0.00 | 31.00 | 0.70 | 0.27 | 12.00 |  |
|  |  | Sweet potato, skin purple, flesh pale-yellow, boiled* (without salt) | 106.00 | 3.00 | 7.00 | 0.00 | 33.00 | 1.40 | 0.37 | 12.00 |  |
|  |  | Yam, tuber, boiled* (without salt) | 109.00 | 3.00 | 16.00 | 0.00 | 29.00 | 0.90 | 0.34 | 50.00 |  |
|  |  | Sweet potato, Komola Sundori, orange flesh, steamed | 87.30 | 582.39 | 5.94 | 0.00 | 25.65 | 0.46 | 0.24 |  | Phytate value unavailable; weight yields and retention factors used |
|  |  | Sweet potato, skin purple, flesh pale-yellow, steamed (without skin) | 93.60 | 2.43 | 5.94 | 0.00 | 27.36 | 1.07 | 0.32 |  | Phytate value unavailable; weight yields and retention factors used |
|  |  | **South/Southeast Asia composite** | 98.00 | 3.00 | 7.00 | 0.00 | 29.00 | 0.80 | 0.32 | 12.00 |  |
| **USDA** | DGLVs | Spinach, raw | 23.00 | 469.00 | 194.00 | 0.00 | 99.00 | 2.71 | 0.53 | 12.01 |  |
|  |  | Kale, cooked, boiled, drained, without salt | 44.00 | 146.00 | 65.00 | 0.00 | 150.00 | 0.84 | 0.27 | 127.00 |  |
|  |  | romaine lettuce, raw | 15.00 | 370.00 | 38.00 | 0.00 | 36.00 | 0.86 | 0.18 | 3.00 |  |
|  |  | Chard, swiss, cooked, boiled, drained, without salt | 20.00 | 306.00 | 9.00 | 0.00 | 58.00 | 2.26 | 0.33 |  | Phytate value unavailable |
|  |  | Pumpkin leaves, cooked, boiled, drained, without salt | 21.00 | 80.00 | 25.00 | 0.00 | 43.00 | 3.20 | 0.20 | 34.00 |  |
|  |  | **USDA composite** | 21.00 | 306.00 | 38.00 | 0.00 | 58.00 | 2.26 | 0.27 | 23.01 |  |
| **Bangladesh** | DGLVs | Indian spinach, boiled* (without salt) | 33.00 | 201.00 | 92.00 | 0.00 | 140.00 | 2.20 | 0.35 |  | Phytate value unavailable |
|  |  | Slender amaranth leaves, boiled* (without salt) | 55.00 | 1030.00 | 50.00 | 0.00 | 255.00 | 4.80 | 1.06 | 38.00 |  |
|  |  | Cabbage, boiled* (without salt) | 27.00 | 5.00 | 24.00 | 0.00 | 35.00 | 0.50 | 0.34 | 5.00 | Used phytate for boiled Chinese cabbage |
|  |  | Spinach, raw | 26.00 | 409.00 | 194.00 | 0.00 | 90.00 | 2.20 | 0.90 | 12.01 |  |
| **Indonesia** | DGLVs | Spinach, cooked | 27.00 | 119.00 | 48.00 | 0.00 | 150.00 | 0.50 | 0.30 |  | Phytate value unavailable |
|  |  | Cabbage, raw | 32.00 | 5.00 | 20.00 | 0.00 | 46.00 | 0.50 | 0.30 | 1.01 |  |
|  |  | Cassava leaf, stir-fried | 68.00 | 456.00 | 12.00 | 0.00 | 166.00 | 1.30 | 2.60 | 28.54 |  |
|  |  | Chinese cabbage, stir-fried | 32.00 | 331.00 | 0.00 | 0.00 | 220.00 | 2.90 | 0.20 | 5.00 |  |
|  |  | **South/Southeast Asia composite** | 32.00 | 266.00 | 36.00 | 0.00 | 145.00 | 1.75 | 0.35 | 8.51 |  |
| **USDA** | VitA-rich fruits and vegetables, excluding DGLVs | Carrots, raw | 41.00 | 835.00 | 19.00 | 0.00 | 33.00 | 0.30 | 0.24 | 19.60 |  |
|  |  | Carrots, cooked, boiled, drained, without salt | 35.00 | 852.00 | 14.00 | 0.00 | 30.00 | 0.34 | 0.20 |  | Phytate value unavailable |
|  |  | Peppers, sweet, red, raw | 26.00 | 157.00 | 46.00 | 0.00 | 7.00 | 0.43 | 0.25 | 17.68 |  |
|  |  | Peppers, sweet, yellow, raw | 27.00 | 10.00 | 26.00 | 0.00 | 11.00 | 0.46 | 0.17 | 14.81 |  |
|  |  | Peppers, sweet, red, sauteed | 133.00 | 138.00 | 2.00 | 0.00 | 7.00 | 0.47 | 0.15 |  | Phytate value unavailable |
|  |  | Pumpkin, cooked, boiled, drained, without salt | 20.00 | 288.00 | 9.00 | 0.00 | 15.00 | 0.57 | 0.23 |  | Phytate value unavailable |
|  |  | Squash, winter, butternut, cooked, baked, WO salt | 40.00 | 558.00 | 19.00 | 0.00 | 41.00 | 0.60 | 0.13 |  | Phytate value unavailable |
|  |  | Mangos, raw | 60.00 | 54.00 | 43.00 | 0.00 | 11.00 | 0.16 | 0.09 | 25.00 |  |
|  |  | Papayas, raw | 43.00 | 47.00 | 37.00 | 0.00 | 20.00 | 0.25 | 0.08 | 25.44 |  |
|  |  | Melon, cantaloupe, raw | 34.00 | 169.00 | 21.00 | 0.00 | 9.00 | 0.21 | 0.18 | 20.00 |  |
|  |  | Guavas, common, raw | 68.00 | 31.00 | 49.00 | 0.00 | 18.00 | 0.26 | 0.23 | 45.89 |  |
|  |  | Passion fruit, (granadilla), purple, raw | 97.00 | 64.00 | 14.00 | 0.00 | 12.00 | 1.60 | 0.10 | 81.40 |  |
|  |  | **USDA composite** | 40.50 | 147.50 | 20.00 | 0.00 | 13.50 | 0.39 | 0.18 | 22.50 |  |
| **Bangladesh** | VitA-rich fruits and vegetables, excluding DGLVs | Carrot, raw | 34.00 | 329.00 | 15.00 | 0.00 | 26.00 | 0.40 | 0.07 | 19.60 |  |
|  |  | Carrot, boiled (without salt) | 43.00 | 364.00 | 9.00 | 0.00 | 34.00 | 0.50 | 0.07 |  | Phytate value unavailable |
|  |  | Pumpkin, boiled (without salt) | 29.00 | 554.00 | 14.00 | 0.00 | 79.00 | 1.10 | 0.14 |  | Phytate value unavailable |
|  |  | Mango, Fazli, orange flesh, ripe, raw | 70.00 | 292.00 | 71.00 | 0.00 | 14.00 | 0.50 | 0.87 | 25.00 |  |
|  |  | Mango, Langra, yellow flesh, ripe, raw | 82.00 | 25.00 | 71.00 | 0.00 | 13.00 | 0.20 | 0.60 | 25.00 |  |
|  |  | Papaya, ripe, raw | 33.00 | 60.00 | 58.00 | 0.00 | 29.00 | 0.30 | 0.17 | 25.44 |  |
|  |  | Melon, Futi, orange flesh, ripe, raw | 17.00 | 105.00 | 21.00 | 0.00 | 17.00 | 0.20 | 0.06 | 20.00 |  |
| **Vietnam** | VitA-rich fruits and vegetables, excluding DGLVs | Carrots, raw | 39.00 | 1686.00 | 19.00 | 0.00 | 43.00 | 0.80 | 1.11 | 19.60 |  |
|  |  | Papaya, ripe | 35.00 | 55.00 | 38.00 | 0.00 | 40.00 | 1.40 | 0.10 | 25.44 |  |
|  |  | Guava, common | 33.00 | 31.00 | 49.00 | 0.00 | 10.00 | 0.90 | 0.50 | 45.89 |  |
|  |  | Mango, common; India mango (ripe) | 69.00 | 38.00 | 14.00 | 0.00 | 10.00 | 0.40 | 0.56 | 25.00 |  |
|  |  | **South/Southeast Asia composite** | 35.00 | 105.00 | 21.00 | 0.00 | 26.00 | 0.50 | 0.17 | 25.00 |  |
| **USDA** | Other fruits, excluding vitA-rich fruits | Bananas, raw | 89.00 | 3.00 | 20.00 | 0.00 | 5.00 | 0.26 | 0.15 | 22.00 |  |
|  |  | Apple, raw | 52.00 | 3.00 | 3.00 | 0.00 | 6.00 | 0.12 | 0.04 | 0.57 |  |
|  |  | Avocados, raw, all commercial varieties | 160.00 | 7.00 | 81.00 | 0.00 | 12.00 | 0.55 | 0.64 | 11.00 |  |
|  |  | Oranges, raw, all commercial varieties | 47.00 | 11.00 | 30.00 | 0.00 | 40.00 | 0.10 | 0.07 | 10.00 |  |
|  |  | Pineapple, raw, all varieties | 50.00 | 3.00 | 18.00 | 0.00 | 13.00 | 0.29 | 0.12 | 8.59 |  |
|  |  | Nuts, coconut meat, raw | 354.00 | 0.00 | 26.00 | 0.00 | 14.00 | 2.43 | 1.10 | 136.00 |  |
|  |  | Watermelon, raw | 30.00 | 28.00 | 3.00 | 0.00 | 7.00 | 0.24 | 0.10 | 10.00 |  |
|  |  | Grapes, red or green (European type, such as Thompson seedless), raw | 69.00 | 3.00 | 2.00 | 0.00 | 10.00 | 0.36 | 0.07 | 0.05 |  |
|  |  | **USDA composite** | 60.50 | 3.00 | 19.00 | 0.00 | 11.00 | 0.28 | 0.11 | 10.00 |  |
| **Bangladesh** | Other fruits, excluding vitA-rich fruits | Apple, with skin, raw | 62.00 | 3.00 | 3.00 | 0.00 | 6.00 | 0.10 | 0.04 | 0.57 |  |
|  |  | Banana, Sagar, ripe, raw | 95.00 | 2.00 | 20.00 | 0.00 | 11.00 | 0.30 | 0.24 | 22.00 |  |
|  |  | Orange, sweet, ripe, raw | 49.00 | 11.00 | 46.00 | 0.00 | 31.00 | 0.10 | 0.07 | 10.00 |  |
|  |  | Lemon, Kagoji, raw | 56.00 | 4.00 | 17.00 | 0.00 | 65.00 | 0.30 | 0.07 | 22.00 |  |
|  |  | Watermelon, ripe, raw | 22.00 | 29.00 | 3.00 | 0.00 | 12.00 | 0.40 | 0.15 | 10.00 |  |
|  |  | Grapes, green, raw | 94.00 | 3.00 | 8.00 | 0.00 | 22.00 | 0.50 | 0.07 | 0.05 |  |
| **Laos** | Other fruits, excluding vitA-rich fruits | Apple, pink, fresh | 63.00 | 7.00 | 3.00 | 0.00 | 7.00 | 0.20 | 0.04 | 0.57 |  |
|  |  | Orange, sweet, fresh | 52.00 | 3.00 | 30.00 | 0.00 | 33.00 | 0.40 | 0.70 | 10.00 |  |
|  |  | Banana, ripe, yellow | 105.00 | 8.00 | 21.00 | 0.00 | 11.00 | 0.40 | 0.10 | 22.00 |  |
|  |  | Banana, ripe, yellow, boiled | 105.00 | 6.00 | 13.00 | 0.00 | 10.00 | 0.40 | 0.10 |  | Phytate value unavailable |
|  |  | **South/Southeast Asia composite** | 62.50 | 5.00 | 15.00 | 0.00 | 11.50 | 0.35 | 0.09 | 10.00 |  |
| **USDA** | Other vegetables, excluding DGLVs and vit A-rich vegetables | Tomatoes, red, ripe, raw, year round average | 18.00 | 42.00 | 15.00 | 0.00 | 10.00 | 0.27 | 0.17 | 2.39 |  |
|  |  | Tomatoes, red, ripe, cooked | 18.00 | 24.00 | 13.00 | 0.00 | 11.00 | 0.68 | 0.14 |  | Phytate value unavailable |
|  |  | Peppers, sweet, green, raw | 20.00 | 18.00 | 10.00 | 0.00 | 10.00 | 0.34 | 0.13 | 15.96 |  |
|  |  | Peppers, sweet, green, cooked, boiled, drained, without salt | 28.00 | 23.00 | 16.00 | 0.00 | 9.00 | 0.46 | 0.12 |  | Phytate value unavailable |
|  |  | Okra, raw | 33.00 | 36.00 | 60.00 | 0.00 | 82.00 | 0.62 | 0.58 | 5.20 |  |
|  |  | Okra, cooked, boiled, drained, without salt | 22.00 | 14.00 | 46.00 | 0.00 | 77.00 | 0.28 | 0.43 | 13.00 |  |
|  |  | Eggplant, cooked, boiled, drained, without salt | 35.00 | 2.00 | 14.00 | 0.00 | 6.00 | 0.25 | 0.12 |  | Phytate value unavailable |
|  |  | Cauliflower, cooked, boiled, drained, without salt | 23.00 | 1.00 | 44.00 | 0.00 | 16.00 | 0.32 | 0.17 |  | Phytate value unavailable |
|  |  | Beans, snap, green, cooked, boiled, drained, without salt | 35.00 | 32.00 | 33.00 | 0.00 | 44.00 | 0.65 | 0.25 |  | Phytate value unavailable |
|  |  | Cucumber, with peel, raw | 15.00 | 5.00 | 7.00 | 0.00 | 16.00 | 0.28 | 0.20 | 17.52 |  |
|  |  | **USDA composite** | 22.50 | 20.50 | 15.50 | 0.00 | 13.50 | 0.33 | 0.17 | 13.00 |  |
| **Bangladesh** | Other vegetables, excluding DGLVs and vit A-rich vegetables | Tomato, red, ripe, raw | 16.00 | 9.00 | 15.00 | 0.00 | 13.00 | 0.20 | 0.41 | 2.39 |  |
|  |  | Tomato, red, ripe, boiled* (without salt) | 30.00 | 16.00 | 15.00 | 0.00 | 24.00 | 0.40 | 0.66 |  | Phytate value unavailable |
|  |  | Okra/ladies finger, raw | 39.00 | 19.00 | 60.00 | 0.00 | 93.00 | 0.90 | 0.34 | 5.20 |  |
|  |  | Okra/ladies finger, boiled* (without salt) | 32.00 | 15.00 | 26.00 | 0.00 | 72.00 | 0.70 | 0.23 | 13.00 |  |
|  |  | Brinjal, purple, long, boiled* (without salt) | 26.00 | 4.00 | 20.00 | 0.00 | 24.00 | 0.40 | 0.52 |  | Phytate value unavailable |
|  |  | Cauliflower, boiled* (without salt) | 28.00 | 1.00 | 32.00 | 0.00 | 36.00 | 0.80 | 0.37 |  | Phytate value unavailable |
|  |  | Cucumber, peeled, raw | 17.00 | 4.00 | 14.00 | 0.00 | 13.00 | 0.60 | 0.17 | 17.52 |  |
| **Laos** | Other vegetables, excluding DGLVs and vit A-rich vegetables | Cucumber, fresh | 23.00 | 5.00 | 9.00 | 0.00 | 20.00 | 0.40 | 0.20 | 17.52 |  |
|  |  | Tomato, fresh | 25.00 | 44.00 | 9.00 | 0.00 | 17.00 | 0.90 | 0.20 | 2.39 |  |
|  |  | **South/Southeast Asia composite** | 26.00 | 9.00 | 15.00 | 0.00 | 24.00 | 0.60 | 0.34 | 9.10 |  |

Supplemental Table 3: First level granular food composition data on animal-source foods (ASFs)

| **Food Group** | **Food (100 g)** | **Country FCT or regional composite FCT** | **kcal** | **Vit A (mcg RAE)** | **Folate (mcg DFE)** | **Vit B_12_ (mcg)** | **Calcium (mg)** | **Iron (mg)** | **Zinc (mg)** | **Comments** |
| --- | --- | --- | --- | --- | --- | --- | --- | --- | --- | --- |
| Eggs | Hen egg | USDA | 155.00 | 149.00 | 44.00 | 1.10 | 50.00 | 1.20 | 1.10 |  |
|  | Hen egg | South/Southeast Asia | 158.00 | 178.00 | 45.00 | 1.10 | 35.00 | 1.80 | 2.54 | Used vit B_12_ from FDC |
|  | Hen egg | **Composite** | 156.50 | 163.50 | 44.50 | 1.10 | 42.50 | 1.50 | 1.82 |  |
| Milk and Dairy Products | Fresh cow milk | USDA | 60.00 | 32.00 | 0.00 | 0.54 | 123.00 | 0.00 | 0.41 |  |
|  | Fresh cow milk | Indonesia | 67.00 | 55.00 | 6.00 | 0.40 | 115.00 | 0.10 | 0.40 |  |
|  | Fresh cow milk | Vietnam | 74.00 | 52.00 | 5.00 | 0.44 | 120.00 | 0.10 | 0.40 |  |
|  | Fresh cow milk | **Composite** | 67.00 | 52.00 | 5.00 | 0.44 | 120.00 | 0.10 | 0.40 |  |
| Milk and Dairy Products | Cooked cow milk | USDA | 61.00 | 46.00 | 5.00 | 0.45 | 113.00 | 0.03 | 0.37 |  |
|  | Cooked cow milk | Bangladesh | 63.00 | 32.00 | 8.50 | 0.45 | 103.00 | 0.10 | 0.45 | Used vit B_12_ from FDC |
|  | Cooked cow milk | Thailand | 58.00 | 29.00 | 4.00 | 0.25 | 113.00 | 0.10 | 0.34 |  |
|  | Cooked cow milk | **Composite** | 61.00 | 32.00 | 5.00 | 0.45 | 113.00 | 0.10 | 0.37 |  |
| Milk and Dairy Products | Fresh goat milk | USDA | 69.00 | 57.00 | 1.00 | 0.07 | 134.00 | 0.05 | 0.30 |  |
|  | Fresh goat milk | Bangladesh | 68.00 | 32.00 | 1.00 | 0.07 | 152.00 | 0.20 | 0.30 | Used vit B_12_ from FDC |
|  | Fresh goat milk | **Composite** | 68.50 | 44.50 | 1.00 | 0.07 | 143.00 | 0.13 | 0.30 |  |
| Milk and Dairy Products | Yoghurt | USDA | 61.00 | 27.00 | 7.00 | 0.37 | 121.00 | 0.05 | 0.59 |  |
|  | Yoghurt | Vietnam | 61.00 | 26.00 | 7.00 | 0.37 | 120.00 | 0.10 | 0.59 |  |
|  | Yoghurt | **Composite** | 61.00 | 26.50 | 7.00 | 0.37 | 120.50 | 0.08 | 0.59 |  |
| Milk and Dairy Products | Cheese | USDA | 353.50 | 203.50 | 9.00 | 1.21 | 606.00 | 0.30 | 3.30 |  |
|  | Cheese | South/Southeast Asia | 363.00 | 245.00 | 29.00 | 0.62 | 775.00 | 0.40 | 3.33 |  |
|  | Cheese | **Composite** | 358.25 | 224.25 | 19.00 | 0.92 | 690.50 | 0.35 | 3.32 |  |
| Ruminant meat | Beef | USDA | 273.25 | 2.75 | 7.25 | 2.26 | 10.00 | 2.52 | 5.30 |  |
|  | Beef | Laos | 170.00 | 2.50 | 2.00 | 1.65 | 9.00 | 3.95 | 6.30 |  |
|  | Beef | Indonesia | 274.00 | 0.00 | 3.00 | 1.60 | 90.00 | 2.50 | 8.40 |  |
|  | Beef | **Composite** | 273.25 | 2.50 | 3.00 | 1.65 | 10.00 | 2.52 | 6.30 |  |
| Ruminant meat | Goat | USDA | 146.00 | 0.00 | 5.00 | 1.18 | 17.00 | 3.69 | 5.21 |  |
|  | Goat | Bangladesh | 78.77 | 0.00 | 2.37 | 1.19 | 6.85 | 1.82 | 2.67 | Used vit B_12_ from FDC |
|  | Goat | **Composite** | 112.39 | 0.00 | 3.69 | 1.19 | 11.93 | 2.76 | 3.94 |  |
| Ruminant meat | Lamb/mutton | USDA | 289.67 | 0.00 | 17.00 | 2.47 | 18.33 | 1.99 | 5.49 |  |
|  | Lamb/mutton | Bangladesh | 130.83 | 4.81 | 2.85 | 2.59 | 7.42 | 1.43 | 2.62 | Used vit B_12_ from FDC |
|  | Lamb/mutton | **Composite** | 210.25 | 2.41 | 9.93 | 2.53 | 12.88 | 1.71 | 4.06 |  |
| Other meat | Pork | USDA | 241.60 | 1.80 | 2.60 | 0.66 | 14.60 | 0.92 | 2.60 |  |
|  | Pork | Laos | 226.50 | 0.00 | 2.50 | 0.62 | 26.50 | 2.00 | 1.54 |  |
|  | Pork | **Composite** | 234.05 | 0.90 | 2.55 | 0.64 | 20.55 | 1.46 | 2.07 |  |
| Poultry | Chicken | USDA | 237.25 | 30.50 | 5.75 | 0.26 | 13.00 | 1.13 | 1.71 |  |
|  | Chicken | Laos | 157.42 | 3.24 | 2.79 | 0.20 | 6.78 | 0.46 | 0.83 |  |
|  | Chicken | **Composite** | 197.34 | 16.87 | 4.27 | 0.23 | 9.89 | 0.80 | 1.27 |  |
| Organ meat | Beef liver | USDA | 181.50 | 8521.00 | 254.50 | 76.22 | 6.00 | 6.31 | 5.23 |  |
|  | Beef liver | Laos | 121.03 | 3810.15 | 196.70 | 44.59 | 14.56 | 8.99 | 3.37 |  |
|  | Beef liver | **Composite** | 151.27 | 6165.58 | 225.60 | 60.41 | 10.28 | 7.65 | 4.30 |  |
| Organ meat | Goat/lam liver | USDA | 229.00 | 7636.50 | 236.50 | 81.10 | 8.50 | 9.24 | 6.76 | Values from USDA FDC only |
| Organ meat | Chicken liver | USDA | 173.50 | 3758.50 | 573.25 | 17.44 | 10.75 | 11.52 | 3.76 |  |
|  | Chicken liver | Laos | 121.00 | 3225.50 | 444.50 | 14.80 | 13.00 | 7.30 | 3.12 |  |
|  | Chicken liver | **Composite** | 147.25 | 3492.00 | 508.88 | 16.12 | 11.88 | 9.41 | 3.44 |  |
| Organ meat | Pork liver | USDA | 165.00 | 5405.00 | 163.00 | 18.67 | 10.00 | 17.92 | 6.72 |  |
|  | Pork liver | Laos | 113.75 | 4442.38 | 135.45 | 15.10 | 10.92 | 13.79 | 4.58 |  |
|  | Pork liver | **Composite** | 139.38 | 4923.69 | 149.23 | 16.89 | 10.46 | 15.86 | 5.65 |  |
| Organ meat | Heart | USDA | 175.00 | 3.50 | 4.50 | 9.05 | 10.50 | 6.11 | 3.39 |  |
|  | Heart | Vietnam | 81.12 | 5.97 | 25.20 | 3.24 | 7.41 | 4.40 | 3.12 |  |
|  | Heart | **Composite** | 128.06 | 4.74 | 14.85 | 6.15 | 8.96 | 5.26 | 3.26 |  |
| Organ meat | Spleen | USDA | 149.00 | 0.00 | 4.00 | 5.02 | 13.00 | 38.70 | 3.54 | Values from USDA FDC only |
| Organ meat | Kidney | USDA | 151.00 | 78.00 | 81.00 | 24.90 | 18.00 | 5.80 | 3.80 |  |
|  | Kidney | Vietnam | 56.70 | 94.50 | 24.99 | 4.46 | 5.60 | 5.32 | 1.64 |  |
|  | Kidney | **Composite** | 103.85 | 86.25 | 53.00 | 14.68 | 11.80 | 5.56 | 2.72 |  |
| Fish and seafood | Fresh fish | USDA | 128.06 | 5.97 | 24.99 | 6.15 | 10.50 | 5.56 | 3.26 |  |
|  | Fresh fish | South/Southeast Asia | 112.84 | 13.00 | 6.00 | 1.86 | 49.72 | 0.72 | 0.91 |  |
|  | Fresh fish | **Composite** | 120.45 | 9.49 | 15.50 | 4.00 | 30.11 | 3.14 | 2.08 |  |
| Fish and seafood | Crustaceans | USDA | 89.00 | 1.43 | 18.00 | 1.00 | 91.00 | 0.29 | 3.81 |  |
|  | Crustaceans | South/Southeast Asia | 95.00 | 4.75 | 11.33 | 1.46 | 82.50 | 2.10 | 2.60 |  |
|  | Crustaceans | **Composite** | 92.00 | 3.09 | 14.67 | 1.23 | 86.75 | 1.20 | 3.21 |  |
| Fish and seafood | Bivalves | USDA | 171.00 | 91.00 | 8.00 | 20.22 | 33.00 | 3.86 | 2.67 |  |
|  | Bivalves | South/Southeast Asia | 40.50 | 45.90 | 6.09 | 15.05 | 103.80 | 4.11 | 1.13 |  |
|  | Bivalves | **Composite** | 105.75 | 68.45 | 7.05 | 17.64 | 68.40 | 3.99 | 1.90 |  |
| Fish and seafood | Canned fish, without bones | USDA | 136.00 | 20.00 | 4.00 | 2.55 | 17.00 | 1.39 | 0.69 | Values from USDA FDC only |
| Fish and seafood | Canned fish, with bones | USDA | 182.00 | 42.50 | 10.00 | 3.91 | 240.00 | 2.48 | 1.17 | Values from USDA FDC only |

Supplemental Table 4: Second level granular food composition database on animal-source foods (ASFs)

| **Country FCT** | **Food group** | **Food (100 g)** | **kcal** | **Vit A (mcg RAE)** | **Folate (mcg DFE)** | **Vit B_12_ (mcg)** | **Calcium (mg)** | **Iron (mg)** | **Zinc (mg)** | **Comments** |
| --- | --- | --- | --- | --- | --- | --- | --- | --- | --- | --- |
| **USDA** | Milk and dairy products | Cheese, cheddar | 408.00 | 316.00 | 21.00 | 1.06 | 707.00 | 0.16 | 3.67 |  |
|  |  | Cottage cheese, farmer's | 143.00 | 83.00 | 11.00 | 0.41 | 78.00 | 0.07 | 0.38 |  |
|  |  | Cheese, mozzarella, whole milk | 299.00 | 179.00 | 7.00 | 2.28 | 505.00 | 0.44 | 2.92 |  |
|  |  | Cheese, parmesan, hard | 421.00 | 228.00 | 6.00 | 1.35 | 884.00 | 0.45 | 4.33 |  |
|  |  | **USDA composite** | 353.50 | 203.50 | 9.00 | 1.21 | 606.00 | 0.30 | 3.30 |  |
| **Bangladesh** | Milk and dairy products | Cheese, cottage, 25% fat | 346.00 | 205.00 | 40.00 | 0.41 | 790.00 | 0.30 | 3.55 | Used vit B**_12_** from USDA |
| **Vietnam** | Milk and dairy products | Cheese, cheddar | 380.00 | 285.00 | 18.00 | 0.83 | 760.00 | 0.50 | 3.11 |  |
|  |  | **South/Southeast Asia composite** | 363.00 | 245.00 | 29.00 | 0.62 | 775.00 | 0.40 | 3.33 |  |
| **USDA** | Ruminant meat | Beef, stew meat, cooked, lean and fat eaten | 236.00 | 0.00 | 9.00 | 2.60 | 5.00 | 3.13 | 4.30 |  |
|  |  | Beef, brisket, whole, separable lean and fat, trimmed to 1/8" fat, all grades, cooked, braised | 331.00 | 0.00 | 7.00 | 2.40 | 7.00 | 2.46 | 5.77 |  |
|  |  | Beef, rib eye steak, boneless, lip-on, separable lean and fat, trimmed to 1/8" fat, all grades, cooked, grilled | 291.00 | 8.00 | 6.00 | 2.10 | 11.00 | 2.24 | 5.91 |  |
|  |  | Beef steak, fried, lean and fat eaten | 235.00 | 3.00 | 7.00 | 1.94 | 17.00 | 2.26 | 5.21 |  |
|  |  | **Beef composite** | 273.25 | 2.75 | 7.25 | 2.26 | 10.00 | 2.52 | 5.30 |  |
| **Laos** | Ruminant meat | Beef, grilled | 190.00 | 3.00 | 3.00 | 2.20 | 9.00 | 4.90 | 7.60 |  |
|  |  | Beef, blanched | 150.00 | 2.00 | 1.00 | 1.10 | 9.00 | 3.00 | 5.00 |  |
|  |  | **Beef composite** | 170.00 | 2.50 | 2.00 | 1.65 | 9.00 | 3.95 | 6.30 |  |
| **Indonesia** | Ruminant meat | Beef, fried | 274.00 | 0.00 | 3.00 | 1.60 | 90.00 | 2.50 | 8.40 |  |
| **USDA** | Ruminant meat | Goat, boiled | 142.00 | 0.00 | 5.00 | 1.18 | 17.00 | 3.70 | 5.23 |  |
|  |  | Game meat, goat, cooked, roasted | 143.00 | 0.00 | 5.00 | 1.19 | 17.00 | 3.73 | 5.27 |  |
|  |  | Goat, fried | 153.00 | 0.00 | 5.00 | 1.16 | 17.00 | 3.64 | 5.14 |  |
|  |  | **Goat composite** | 146.00 | 0.00 | 5.00 | 1.18 | 17.00 | 3.69 | 5.21 |  |
| **Bangladesh** | Ruminant meat | Goat meat, lean, boiled | 70.80 | 0.00 | 1.80 | 1.18 | 5.76 | 1.68 | 2.40 | Used weight yields and nutrient retention factors; used vit B**_12_** values from USDA |
|  |  | Goat meat, lean, roasted | 86.73 | 0.00 | 2.94 | 1.19 | 7.94 | 1.96 | 2.94 | Used weight yields and nutrient retention factors; used vit B**_12_** values from USDA |
|  |  | **Goat composite** | 78.77 | 0.00 | 2.37 | 1.19 | 6.85 | 1.82 | 2.67 |  |
| **USDA** | Ruminant meat | Lamb, roast, cooked, lean and fat eaten | 265.00 | 0.00 | 20.00 | 2.59 | 16.00 | 1.96 | 4.78 |  |
|  |  | Lamb, foreshank, separable lean and fat, trimmed to 1/4" fat, choice, cooked, braised | 243.00 | 0.00 | 17.00 | 2.28 | 20.00 | 2.14 | 7.69 |  |
|  |  | Lamb, rib, separable lean and fat, trimmed to 1/4'' fat, choice, cooked, broiled | 361.00 | 0.00 | 14.00 | 2.54 | 19.00 | 1.88 | 4.00 |  |
|  |  | **Lamb/Mutton composite** | 289.67 | 0.00 | 17.00 | 2.47 | 18.33 | 1.99 | 5.49 |  |
| **Bangladesh** | Ruminant meat | Lamb/mutton, meat, moderately fat, roasted | 144.06 | 5.29 | 3.53 | 2.59 | 8.60 | 1.54 | 2.88 | Used weight yields and nutrient retention factors; used vit B**_12_** values from USDA |
|  |  | Lamb/mutton, meat, moderately fat, boiled | 117.60 | 4.32 | 2.16 |  | 6.24 | 1.32 | 2.35 | Used weight yields and nutrient retention factors |
|  |  | **Lamb/Mutton composite** | 130.83 | 4.81 | 2.85 | 2.59 | 7.42 | 1.43 | 2.62 |  |
| **USDA** | Poultry | Chicken, stewing, meat and skin, cooked, stewed | 285.00 | 39.00 | 5.00 | 0.23 | 13.00 | 1.37 | 1.77 |  |
|  |  | Chicken, broilers or fryers, meat and skin, cooked, roasted | 239.00 | 48.00 | 5.00 | 0.30 | 15.00 | 1.26 | 1.94 |  |
|  |  | Chicken breast, grilled, WO sauce, skin eaten | 206.00 | 17.00 | 6.00 | 0.17 | 7.00 | 0.53 | 0.87 |  |
|  |  | Chicken, broilers or fryers, meat only, cooked, fried | 219.00 | 18.00 | 7.00 | 0.34 | 17.00 | 1.35 | 2.24 |  |
|  |  | **Chicken composite** | 237.25 | 30.50 | 5.75 | 0.26 | 13.00 | 1.13 | 1.71 |  |
| **Laos** | Poultry | Chicken, boiled | 193.00 | 5.00 | 4.00 | 0.24 | 7.00 | 0.60 | 1.00 |  |
|  |  | Chicken, roasted | 121.83 | 1.47 | 1.57 | 0.15 | 6.55 | 0.31 | 0.66 | Used weight yields and nutrient retention factors |
|  |  | **Chicken composite** | 157.42 | 3.24 | 2.79 | 0.20 | 6.78 | 0.46 | 0.83 |  |
| **USDA** | Other meat | Pork roast, loin, cooked, lean and fat eaten | 246.00 | 3.00 | 6.00 | 0.70 | 19.00 | 0.98 | 2.30 |  |
|  |  | Pork, fresh, shoulder, (Boston butt), blade (steaks), separable lean and fat, cooked, braised | 267.00 | 2.00 | 0.00 | 0.93 | 26.00 | 1.75 | 4.84 |  |
|  |  | Pork, fresh, loin, center rib (chops), boneless, separable lean and fat, cooked, pan-fried | 273.00 | 2.00 | 7.00 | 0.59 | 11.00 | 0.73 | 2.04 |  |
|  |  | Pork chop, fried, lean and fat eaten | 211.00 | 1.00 | 0.00 | 0.64 | 8.00 | 0.50 | 1.92 |  |
|  |  | Pork chop, stewed, lean and fat eaten | 211.00 | 1.00 | 0.00 | 0.43 | 9.00 | 0.63 | 1.92 |  |
|  |  | **Pork composite** | 241.60 | 1.80 | 2.60 | 0.66 | 14.60 | 0.92 | 2.60 |  |
| **Laos** | Other meat | Pork, boiled | 204.00 | 0.00 | 2.00 | 0.58 | 10.00 | 1.50 | 1.30 |  |
|  |  | Pork, grilled | 249.00 | 0.00 | 3.00 | 0.65 | 43.00 | 2.50 | 1.78 |  |
|  |  | **Pork composite** | 226.50 | 0.00 | 2.50 | 0.62 | 26.50 | 2.00 | 1.54 |  |
| **USDA** | Organ meat | Beef liver, braised | 189.00 | 9363.00 | 251.00 | 69.99 | 6.00 | 6.49 | 5.26 |  |
|  |  | Beef liver, fried | 174.00 | 7679.00 | 258.00 | 82.44 | 6.00 | 6.12 | 5.19 |  |
|  |  | **Beef liver composite** | 181.50 | 8521.00 | 254.50 | 76.22 | 6.00 | 6.31 | 5.23 |  |
| **Laos** | Organ meat | Beef, liver, grilled | 133.00 | 3841.00 | 185.00 | 51.60 | 16.00 | 10.10 | 3.87 |  |
|  |  | Beef, liver, pan fried | 109.06 | 3779.30 | 208.40 | 37.58 | 13.12 | 7.87 | 2.86 | Used weight yields and nutrient retention factors |
|  |  | **Beef liver composite** | 121.03 | 3810.15 | 196.70 | 44.59 | 14.56 | 8.99 | 3.37 |  |
| **USDA** | Organ meat | Lamb, variety meats and by-products, liver, cooked, braised | 220.00 | 7491.00 | 73.00 | 76.50 | 8.00 | 8.28 | 7.89 |  |
|  |  | Lamb, variety meats and by-products, liver, cooked, pan-fried | 238.00 | 7782.00 | 400.00 | 85.70 | 9.00 | 10.20 | 5.63 |  |
|  |  | **Goat/lamb liver composite** | 229.00 | 7636.50 | 236.50 | 81.10 | 8.50 | 9.24 | 6.76 |  |
| **USDA** | Organ meat | Chicken liver, braised | 166.00 | 3948.00 | 573.00 | 16.71 | 11.00 | 11.53 | 3.95 |  |
|  |  | Chicken liver, fried | 189.00 | 2809.00 | 582.00 | 15.07 | 11.00 | 10.04 | 3.08 |  |
|  |  | Chicken, liver, all classes, cooked, simmered | 167.00 | 3981.00 | 578.00 | 16.85 | 11.00 | 11.63 | 3.98 |  |
|  |  | Chicken, liver, all classes, cooked, pan-fried | 172.00 | 4296.00 | 560.00 | 21.13 | 10.00 | 12.88 | 4.01 |  |
|  |  | **Chicken liver composite** | 173.50 | 3758.50 | 573.25 | 17.44 | 10.75 | 11.52 | 3.76 |  |
| **Laos** | Organ meat | Chicken liver, boiled | 121.00 | 3178.00 | 462.00 | 13.50 | 13.00 | 7.30 | 3.18 |  |
|  |  | Chicken liver, grilled | 121.00 | 3273.00 | 427.00 | 16.10 | 13.00 | 7.30 | 3.06 |  |
|  |  | **Chicken liver composite** | 121.00 | 3225.50 | 444.50 | 14.80 | 13.00 | 7.30 | 3.12 |  |
| **USDA** | Organ meat | Pork, fresh, variety meats and by-products, liver, cooked, braised | 165.00 | 5405.00 | 163.00 | 18.67 | 10.00 | 17.92 | 6.72 |  |
| **Laos** | Organ meat | Pork, liver, grilled | 125.00 | 4242.00 | 128.00 | 14.70 | 12.00 | 15.50 | 5.27 |  |
|  |  | Pork liver, pan fried | 102.50 | 4642.76 | 142.89 | 15.50 | 9.84 | 12.07 | 3.88 | Used weight yields and nutrient retention factors |
|  |  | **Pork liver composite** | 113.75 | 4442.38 | 135.45 | 15.10 | 10.92 | 13.79 | 4.58 |  |
| **USDA** | Organ meat | Chicken, heart, all classes, cooked, simmered | 185.00 | 8.00 | 80.00 | 7.29 | 19.00 | 9.03 | 7.30 |  |
|  |  | Lamb, variety meats and by-products, heart, cooked, braised | 185.00 | 0.00 | 2.00 | 11.20 | 14.00 | 5.52 | 3.68 |  |
|  |  | Beef, variety meats and by-products, heart, cooked, simmered | 165.00 | 0.00 | 5.00 | 10.80 | 5.00 | 6.38 | 2.87 |  |
|  |  | Pork, fresh, variety meats and by-products, heart, cooked, braised | 148.00 | 7.00 | 4.00 | 3.79 | 7.00 | 5.83 | 3.09 |  |
|  |  | **Heart composite** | 175.00 | 3.50 | 4.50 | 9.05 | 10.50 | 6.11 | 3.39 |  |
| **Vietnam** | Organ meat | Chicken heart, pan fried | 88.92 | 6.32 | 47.74 | 4.26 | 9.36 | 4.42 | 4.37 | Used weight yields and nutrient retention factors |
|  |  | Pig heart, pan fried | 73.32 | 5.62 | 2.65 | 2.22 | 5.46 | 4.37 | 1.86 | Used weight yields and nutrient retention factors |
|  |  | **Heart composite** | 81.12 | 5.97 | 25.20 | 3.24 | 7.41 | 4.40 | 3.12 |  |
| **USDA** | Organ meat | Beef, variety meats and by-products, spleen, cooked, braised | 145.00 | 0.00 | 4.00 | 5.02 | 12.00 | 39.40 | 2.79 |  |
|  |  | Lamb, variety meats and by-products, spleen, cooked, braised | 156.00 | 0.00 | 4.00 | 5.29 | 13.00 | 38.70 | 3.94 |  |
|  |  | Pork, fresh, variety meats and by-products, spleen, cooked, braised | 149.00 | 0.00 | 4.00 | 2.76 | 13.00 | 22.20 | 3.54 |  |
|  |  | **Spleen composite** | 149.00 | 0.00 | 4.00 | 5.02 | 13.00 | 38.70 | 3.54 |  |
| **USDA** | Organ meat | Pork, fresh, variety meats and by-products, kidneys, cooked, braised | 151.00 | 78.00 | 41.00 | 7.79 | 13.00 | 5.29 | 4.15 |  |
|  |  | Lamb, variety meats and by-products, kidneys, cooked, braised | 137.00 | 137.00 | 81.00 | 78.90 | 18.00 | 12.40 | 3.80 |  |
|  |  | Beef, variety meats and by-products, kidneys, cooked, simmered | 158.00 | 0.00 | 83.00 | 24.90 | 19.00 | 5.80 | 2.84 |  |
|  |  | **Kidney composite** | 151.00 | 78.00 | 81.00 | 24.90 | 18.00 | 5.80 | 3.80 |  |
| **Vietnam** | Organ meat | Pork kidney, pan fried | 56.70 | 94.50 | 24.99 | 4.46 | 5.60 | 5.32 | 1.64 | Used weight yields and nutrient retention factors |
| **USDA** | Fresh fish | Sea bass, baked or broiled, no fat added | 122.00 | 52.00 | 6.00 | 0.34 | 13.00 | 0.37 | 0.50 |  |
|  |  | Sea bass, steamed or poached | 122.00 | 46.00 | 5.00 | 0.32 | 13.00 | 0.36 | 0.50 |  |
|  |  | Tilapia, baked or broiled, no fat added | 121.00 | 0.00 | 27.00 | 1.79 | 13.00 | 0.71 | 0.42 |  |
|  |  | Tilapia, steamed or poached | 121.00 | 0.00 | 24.00 | 1.69 | 13.00 | 0.70 | 0.42 |  |
|  |  | Herring, baked or broiled, no fat added | 199.00 | 30.00 | 12.00 | 14.63 | 72.00 | 1.39 | 1.25 |  |
|  |  | Carp, baked or broiled, no fat added | 160.00 | 10.00 | 17.00 | 1.64 | 52.00 | 1.56 | 1.86 |  |
|  |  | Carp, steamed or poached | 160.00 | 10.00 | 15.00 | 1.64 | 52.00 | 1.56 | 1.86 |  |
|  |  | **USDA composite** | 122.00 | 10.00 | 15.00 | 1.64 | 13.00 | 0.71 | 0.50 |  |
| **Laos** | Fresh fish | Short bodied mackarel fried | 236.00 | 26.00 | 2.00 | 4.84 | 114.00 | 2.40 | 1.00 |  |
|  |  | Short bodied mackarel roasted | 122.00 | 27.00 | 1.00 | 5.70 | 62.00 | 1.40 | 0.60 |  |
|  |  | Nile tilapia roasted | 128.00 | 0.00 | 6.00 | 1.86 | 14.00 | 0.69 | 0.41 |  |
| **Vietnam** | Fresh fish | Carp, bighead, boiled | 103.68 | 0.00 | 7.56 | 0.88 | 37.44 | 0.71 | 1.07 | Used weight yields and nutrient retention factors |
|  |  | Tilapia, stewed | 66.00 | 0.00 | 6.93 | 0.81 | 32.80 | 0.28 | 0.98 | Used weight yields and nutrient retention factors |
| **Bangladesh** | Fresh fish | Koi (climbing perch), indigenous, eyes included, boiled | 97.50 | 112.88 |  |  | 307.50 | 0.72 | 0.85 | Used weight yields and nutrient retention factors |
|  |  | **South/Southeast Asia composite** | 112.84 | 13.00 | 6.00 | 1.86 | 49.72 | 0.72 | 0.91 |  |
| **USDA** | Crustaceans | Crustaceans, crab, blue, cooked, moist heat | 83.00 | 1.00 | 51.00 | 3.33 | 91.00 | 0.50 | 3.81 |  |
|  |  | Crustaceans, lobster, northern, cooked, moist heat | 89.00 | 1.43 | 11.00 | 1.00 | 96.00 | 0.29 | 4.05 |  |
|  |  | Shrimp, steamed or boiled | 91.00 | 62.00 | 18.00 | 0.85 | 69.00 | 0.24 | 1.24 |  |
|  |  | **USDA composite** | 89.00 | 1.43 | 18.00 | 1.00 | 91.00 | 0.29 | 3.81 |  |
| **Vietnam** | Crustaceans | Shrimp, sea water, boiled | 53.30 | 11.70 | 1.37 | 0.60 | 51.35 | 0.83 | 0.72 | Used weight yields and nutrient retention factors |
|  |  | Shrimp, fields river, boiled | 58.50 | 8.78 | 8.65 | 0.58 | 47.45 | 1.14 | 0.63 | Used weight yields and nutrient retention factors |
|  |  | **South/Southeast Asia composite** | 55.90 | 10.24 | 5.01 | 0.59 | 49.40 | 0.99 | 0.68 |  |
| **USDA** | Bivalves | Mollusks, mussel, blue, cooked, moist heat | 172.00 | 91.00 | 76.00 | 24.00 | 33.00 | 6.72 | 2.67 |  |
|  |  | Clams, steamed or boiled | 171.00 | 161.00 | 7.00 | 20.22 | 78.00 | 2.90 | 1.02 |  |
|  |  | Ostrich, oyster, raw | 125.00 | 0.00 | 8.00 | 4.91 | 6.00 | 3.86 | 3.59 |  |
|  |  | **USDA composite** | 171.00 | 91.00 | 8.00 | 20.22 | 33.00 | 3.86 | 2.67 |  |
| **Vietnam** | Bivalves | Mussels, fresh water, boiled | 22.80 | 48.60 | 6.72 | 23.71 | 15.00 | 0.72 | 0.82 | Used weight yields and nutrient retention factors |
| **Indonesia** | Bivalves | Clams, boiled | 58.20 | 43.20 | 5.46 | 6.38 | 192.60 | 7.49 | 1.44 | Used weight yields and nutrient retention factors |
|  |  | **South/Southeast Asia composite** | 40.50 | 45.90 | 6.09 | 15.05 | 103.80 | 4.11 | 1.13 |  |
| **USDA** | Canned fish, without bones | Fish, tuna, light, canned in oil, drained solids | 198.00 | 23.00 | 5.00 | 2.20 | 13.00 | 1.39 | 0.90 |  |
|  |  | Fish, tuna, light, canned in water, drained solids | 86.00 | 17.00 | 4.00 | 2.55 | 17.00 | 1.63 | 0.69 |  |
|  |  | Fish, Salmon, pink, canned, drained solids, without skin and bones | 136.00 | 20.00 | 4.00 | 4.96 | 60.00 | 0.57 | 0.65 |  |
|  |  | **USDA composite** | 136.00 | 20.00 | 4.00 | 2.55 | 17.00 | 1.39 | 0.69 |  |
| **USDA** | Canned fish, with bones | Fish, salmon, sockeye, canned, without salt, drained solids with bone | 153.00 | 53.00 | 10.00 | 0.30 | 239.00 | 1.06 | 1.02 |  |
|  |  | Fish, anchovy, european, canned in oil, drained solids | 210.00 | 12.00 | 13.00 | 0.88 | 232.00 | 4.63 | 2.44 |  |
|  |  | Fish, mackerel, jack, canned, drained solids | 156.00 | 130.00 | 5.00 | 6.94 | 241.00 | 2.04 | 1.02 |  |
|  |  | Fish, sardine, Atlantic, canned in oil, drained solids with bone | 208.00 | 32.00 | 10.00 | 8.94 | 382.00 | 2.92 | 1.31 |  |
|  |  | **USDA composite** | 182.00 | 42.50 | 10.00 | 3.91 | 240.00 | 2.48 | 1.17 |  |
